# Macrophage migration inhibitory factor is regulated by HIF-1α and cAMP and promotes renal cyst cell proliferation in a macrophage-independent manner

**DOI:** 10.1007/s00109-020-01964-1

**Published:** 2020-09-04

**Authors:** Wajima Safi, Andre Kraus, Steffen Grampp, Johannes Schödel, Bjoern Buchholz

**Affiliations:** 1 Department of Nephrology and Hypertension, Friedrich-Alexander-University Erlangen-Nuernberg, Ulmenweg 18, D - 91054 Erlangen, Germany; 2 Pluripotency for Organ Regeneration, Institute for Bioengineering of Catalonia (IBEC), The Barcelona Institute of Technology (BIST), Barcelona, Spain

**Keywords:** Polycystic kidney disease, Macrophage migration inhibitory factor, Hypoxia-inducible factor 1α, cAMP

## Abstract

**Abstract:**

Progressive cyst growth leads to decline of renal function in polycystic kidney disease. Macrophage migration inhibitory factor (MIF) was found to be upregulated in cyst-lining cells in a mouse model of polycystic kidney disease and to promote cyst growth. In addition, MIF can be secreted by tubular cells and may contribute to cyst growth in an autocrine manner. However, the underlying mechanisms leading to induction of MIF in cyst-lining cells remained elusive. Here, we demonstrate that hypoxia-inducible transcription factor (HIF) 1α upregulates MIF in cyst-lining cells in a tubule-specific PKD1 knockout mouse. Pharmacological stabilization of HIF-1α resulted in significant increase of MIF in cyst epithelial cells whereas tubule-specific knockout of HIF-1α prevented MIF upregulation. Identical regulation could be found for ABCA1, which has been shown to act as a transport protein for MIF. Furthermore, we show that MIF and ABCA1 are direct target genes of HIF-1α in human primary tubular cells. Next to HIF-1α and hypoxia, we found MIF being additionally regulated by cAMP which is a strong promotor of cyst growth. In line with these findings, HIF-1α- and cAMP-dependent in vitro cyst growth could be decreased by the MIF-inhibitor ISO-1 which resulted in reduced cyst cell proliferation. In conclusion, HIF-1α and cAMP regulate MIF in primary tubular cells and cyst-lining epithelial cells, and MIF promotes cyst growth in the absence of macrophages. In line with these findings, the MIF inhibitor ISO-1 attenuates HIF-1α- and cAMP-dependent in vitro cyst enlargement.

**Key messages:**

• MIF is upregulated in cyst-lining cells in a polycystic kidney disease mouse model.

• MIF upregulation is mediated by hypoxia-inducible transcription factor (HIF) 1α.

• ABCA1, transport protein for MIF, is also regulated by HIF-1α in vitro and in vivo.

• MIF is additionally regulated by cAMP, a strong promotor of cyst growth.

• MIF-inhibitor ISO-1 reduces HIF-1α- and cAMP-dependent cyst growth.

**Electronic supplementary material:**

The online version of this article (10.1007/s00109-020-01964-1) contains supplementary material, which is available to authorized users.

## Introduction

Autosomal dominant polycystic kidney disease (ADPKD) is the most common potentially lethal monogenic disorder affecting approximately 1:1000 [1]. ADPKD is mainly characterized by the development of fluid-filled cysts originating from tubular epithelial cells in both kidneys. Due to continuous cyst enlargement, adjacent intact nephrons become compressed which leads to decline of renal function and, depending on severity, consecutive need for renal replacement therapy [2]. ADPKD is caused by mutations in the PKD1- (~ 85% of cases) or PKD2-gene (~ 15% of cases) [1]. Cyst growth and disease progression have been attributed to several mechanisms, including cyst cell proliferation, transepithelial fluid transport into the cysts’ lumina, macrophage-dependent inflammation, and extracellular matrix deposition [3–6]. Cyst enlargement has been associated with interstitial inflammation reflected by macrophage infiltration resulting in decline of renal function [7, 8]. Inflammation may not only be a secondary event induced during the course of cyst expansion but may also actively contribute to cyst growth [9]. Thus macrophage depletion in an ADPKD mouse model resulted in smaller cysts, less cyst cell proliferation, and improved renal function [7, 8].

One of the pivotal regulators of innate immunity, the cytokine macrophage migration inhibitory factor (MIF), is an integral component of stress response that promotes proinflammatory functions of immune cells [10]. Recently, it has been shown that upregulation of MIF in cyst epithelial cells of an ADPKD mouse model resulted in increased cyst growth [9]. Data obtained from urinary bladder carcinoma cells, renal cancer cells, and in vitro renal tubule cells suggest that MIF may not only unfold its effects by signaling pathways that are induced by attracted macrophages but may also directly promote epithelial cell proliferation [9, 11, 12]. However, the mechanisms leading to induction of MIF in renal epithelial and cyst-lining cells as well as its transport into the extracellular milieu have remained elusive so far.

Continuous cyst enlargement leads to progressive tissue hypoxia which induces HIF-1α in cyst epithelial cells [13–15]. HIF is a heterodimer consisting of a constitutively expressed β-subunit (HIF-β) and one of two alternative, oxygen-dependent α-subunits (HIF-1α and HIF-2α) which in the absence of oxygen accumulate and activate genes involved in metabolism, angiogenesis, immune reactions, and cell proliferation [16, 17]. The continuous degradation of HIF-α by prolyl hydroxylase domain (PHD) enzymes in the presence of oxygen can be inhibited by prolyl-hydroxylase inhibitors which have been approved as erythropoiesis-stimulating agents [17–19]. However, recently, we have shown that stimulation of HIF-1α (the isoform present in tubular and cyst-lining cells) may be detrimental by significantly promoting cyst growth in an ADPKD mouse model [15]. HIF-1α promoted calcium-activated chloride secretion but was also associated with increased cyst cell proliferation, especially in a rapid progressive ADPKD mouse model [14, 15]. Since cyst expansion aggravates regional hypoxia which then induces HIF-1α, a feedforward loop is established that accelerates cyst expansion and disease progression [20]. MIF has been shown to be upregulated by hypoxia in different tumors [21, 22]. In addition, MIF has been identified as a HIF target gene in cells of the trachea, lungs, and in cancer cells [23–26]. A validation of this finding in the context of renal tissue and cysts is lacking. In addition, in vivo MIF–mediated cyst growth has been referred to macrophage recruitment and the release of monocyte chemotactic protein 1 (MCP-1) and inflammatory cytokine TNF-α [9]. However, in vitro MIF may affect proliferation of renal tubule cells and renal clear cell carcinoma cells independent from the presence of macrophages [12, 27]. Therefore, we wondered if secreted MIF may increase cyst growth in an autocrine/paracrine way. Of note, MIF secretion by immune and epithelial cells occurs apically by a nonclassical export route involving ABCA1 transporter [28].

Here, we provide data showing that (i) MIF as well as its transporter protein ABCA1 are regulated by HIF-1α in cyst-lining cells in vivo and in vitro, (ii) that cAMP is also a regulator of MIF, and (iii) MIF induces cyst cell proliferation in vitro in a macrophage-independent way which can be inhibited by the MIF-inhibitor ISO-1.

## Results

### HIF-1α stabilization leads to an increase in MIF and ABCA1 expression in cyst-lining cells in vivo

**Fig. 1:**
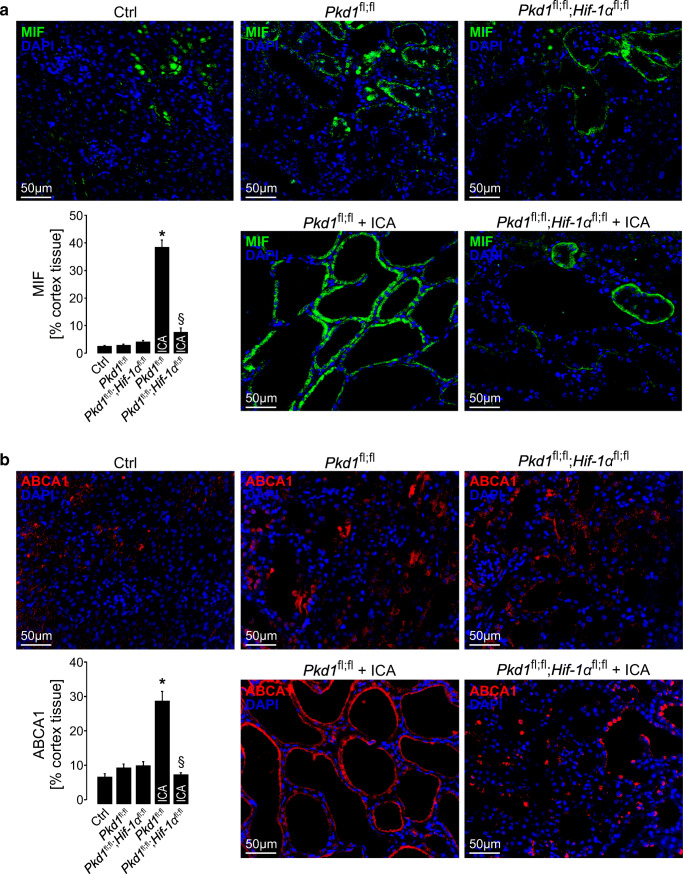
MIF and ABCA1 are expressed in a HIF-1α-dependent manner in cyst-lining cells of an ADPKD mouse model. Tamoxifen was applied at postnatal days 35–37 to induce tubule-specific deletion of PKD1 in Ksp*CreER*^T2^;*Pkd1*^lox;lox^ (*Pkd1*^fl;fl^; *n* = 7) mice. In parallel, genetic deletion was induced in Ksp*CreER*^T2^;*Pkd1*^lox;lox^;Hif-1α^lox/lox^ (*Pkd1*^fl;fl^;*Hif-1α*^fl;fl^; *n* = 5) mice to receive tubular codeletion of PKD1 and HIF-1α. Mice were then either treated with the prolylhydroxylase inhibitor 2-(1-chloro-4-hydroxyisoquinoline-3-carboxamido) acetate (*Pkd1*^fl;fl^ + ICA; *n* = 6); (*Pkd1*^fl;fl^;*Hif-1α*^fl;fl^ + ICA; *n* = 6) or its vehicle for 12 weeks. Noninduced mice served as controls (Ctrl; *n* = 4). **a** As shown previously, the abovementioned ADPKD mouse model (*Pkd1*^fl;fl^) shows a mild progression which does not lead to hypoxia or induction of HIF-1α. In line with these findings, MIF expression did not differ in the cortex between Ctrl, *Pkd1*^fl;fl^, and *Pkd1*^fl;fl^;*Hif-1α*^fl;fl^ kidneys. However, application of ICA (*Pkd1*^fl;fl^ + ICA) resulted in a significant increase of HIF-1α shown previously which was prevented in mice co-deleted for HIF-1α (*Pkd1*^fl;fl^;*Hif-1α*^fl;fl^ + ICA). In line with the assumption of MIF being regulated by HIF-1α, MIF expression was significantly increased in the cortex of *Pkd1*^fl;fl^ + ICA mice which could be prevented in mice codeleted for HIF-1α (*Pkd1*^fl;fl^;*Hif-1α*^fl;fl^ + ICA). **b** ABCA1 shows a comparable pattern of HIF-dependent expression as MIF in cyst cells in the renal cortex of the chosen models. *Significant compared with Ctrl. §Significant compared with *Pkd1*^fl;fl^ + ICA

In order to investigate whether there is evidence for HIF-1α-mediated MIF-expression in vivo, we used an inducible, kidney-epithelium-specific PKD1 knockout mouse model (Ksp*CreER*^T2^;*Pkd1*^lox;lox^) and crossed it with floxed HIF-1α mice (*Hif-1α*^lox;lox^) to obtain an inducible tubule-specific deletion of PKD1 and HIF-1α in renal tubular cells. Tubular deletion of PKD1 was induced at postnatal days 35–37 which resulted in a slowly progressing polycystic renal phenotype which did not result in hypoxia or consecutive induction of HIF-1α as shown previously [15]. However, tubular HIF-1α was significantly induced by treatment with the prolyl-hydroxylase-inhibitor 2-(1-chloro-4-hydroxy-isoquinoline-3-carboxamido) acetate (ICA) [15, 29]. Induction of HIF-1α by ICA resulted in a significant cyst progression which could be rescued by tubular deletion of HIF-1α as shown previously [15, 30]. We tested renal cysts within kidney tissue obtained from the named mouse model for ICA- and HIF-1α-dependent effects on MIF as well as ABCA1 expression by immunohistochemistry. We found a strong induction of MIF by ICA in Ksp*CreER*^T2^;*Pkd1*^lox;lox^ kidneys which was significantly reduced in animals with an additional deletion of tubular HIF-1α (Fig. 1a and Supplemental Figure 1A). ICA also increased MIF expression in wildtype kidneys pointing towards HIF-mediated induction irrespective of the genetic background (Supplemental Figure 2A). In addition, ABCA1 showed a concordant pattern of expression in cysts obtained from the named mouse models (Fig. 1b and Supplemental Figures 1B and 2B). Accordingly, ABCA1 expression was significantly higher in Ksp*CreER*^T2^;*Pkd1*^lox;lox^ kidneys upon treatment with ICA whereas expression was significantly reduced in Ksp*CreER*^T2^;*Pkd1*^lox;lox^;Hif-1α^lox/lox^ mice treated with ICA. MIF expression did not only increase in overall levels but also showed different subcellular patterns which correlated with the degree of cyst formation and expansion. While MIF showed a predominant nuclear pattern in normal and mildly dilated tubules, it showed a switch towards a cytoplasmic localization in cysts (Supplemental Figures 3 and 4). Previous findings suggested that MIF secretion is mediated by ABCA1 [28]. This is further supported by our findings showing coexpression of MIF and ABCA1 in cyst-lining cells (Supplemental Figure 4) and correlating numbers of MIF- and ABCA1-positive cysts in kidneys with different genetic backgrounds and upon treatment with ICA (Supplemental Figure 5). In addition, protein expression of MIF was not restricted to specific tubule-segments but rather correlated with coexpression of HIF-1α (Supplemental Figure 6). These data suggest a HIF-1α-dependent regulation of MIF and ABCA1 in cyst epithelial cells in vivo.

### MIF and ABCA1 are target genes of HIF-1α in human renal epithelial cells

**Fig. 2:**
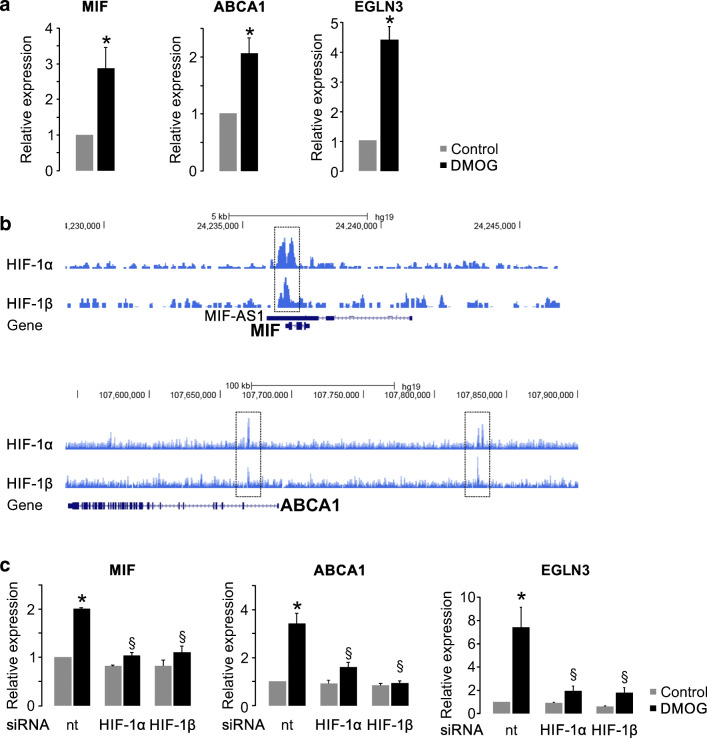
MIF and ABCA1 are HIF-1α target genes in human primary tubular epithelial cells (hPTECs). **a** HIF stabilization by DMOG in hPTCs for 16 h led to a significantly increased expression of MIF and ABCA1 mRNA (*n* = 13 for MIF, *n* = 8 for ABCA1, and *n* = 6 for EGLN3, egl nine homolog 3) compared with vehicle control. The known HIF-1α target gene EGLN3 served as positive control. **b** HIF-1α- and HIF-1β ChIP-seq signals at the genomic regions coding for MIF or ABCA1, respectively, reveal HIF DNA interactions at the promoter of MIF and at two regions at the ABCA1 locus (dotted rectangles) in hPTCs. MIF-AS1: MIF antisense 1 which is encoded close to MIF in the indicated genomic interval of the human genome. **c** Expression qPCR of MIF and ABCA1, respectively, in HIF-1α and HIF-1β siRNA depleted hPTCs with or without DMOG treatment for 16 h. MIF or ABCA1 mRNA levels increased in DMOG-treated cells and are significantly reduced in HIF-depleted cells. EGLN3 served as positive control (*n* = 3 individual experiments). *Significant compared with vehicle control. §Significant compared with cells treated with nontargeting (nt) siRNA

Next, we aimed to evaluate HIF-1α-dependent regulation of MIF and ABCA1 in human primary renal tubular epithelial cells (hPTECs). HPTECs were isolated from healthy parts of the kidneys of patients who were nephrectomized because of kidney cancer [31]. HIF stabilization by dimethyloxalylglycine (DMOG) in hPTECs resulted in increased MIF and ABCA1 mRNA expression (Fig. 2a). To assess the direct involvement of HIF-1α in transcriptional activation of these genes, we interrogated available next-generation sequencing data sets from HIF chromatin immunoprecipitation (ChIP-seq) experiments in hPTECs [32]. We observed HIF DNA-binding (HIF-1α and HIF-1ß) at the promoter region of the MIF gene and at two regions at the ABCA1 locus (Fig. 2b). To confirm involvement of HIF in regulation of MIF and ABCA1 expression, we performed knockdown of HIF-1α and its dimerization partner HIF-1ß in the primary cells (Fig. 2c). Knockdown of either subunit led to a reduced induction of MIF, ABCA1, and the well-characterized HIF-1α target gene EGLN3, which served as control (Fig. 2c). Knockdown of HIF-1α and HIF-1β was also confirmed on protein level (Supplemental Figure 7). In summary, these data show that MIF and ABCA1 are direct transcriptional targets of HIF-1α in human primary renal tubular cells.

### MIF is expressed in plMDCK cysts and upregulated by ICA, hypoxia and forskolin

**Fig. 3:**
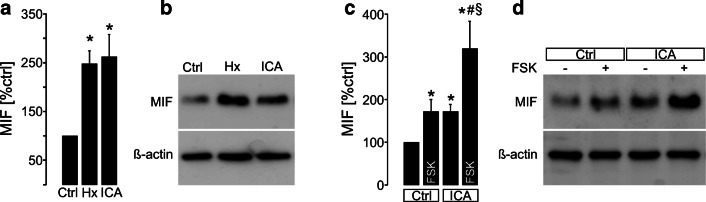
MIF is regulated by HIF-1α and cAMP in cyst-forming plMDCK cells. A Analysis of MIF protein expression normalized to ß-actin in plMDCK cells by Western blot under control condition (Ctrl), in the presence of hypoxia (5%O_2_; Hx) or stimulated with 10 μM ICA (n = 4 individual experiments). **B** Representative Western blot. **C** Analysis of MIF protein expression normalized to ß-actin in plMDCK cells by Western blot under control condition (Ctrl) ± forskolin (FSK; 10 μM) or stimulated with 10 μM ICA ± forskolin (FSK; 10 μM) (n = 7 individual experiments). **D** Representative Western blot. * significant compared to Ctrl (-FSK). § significant compared to ICA (-FSK). # significant compared to Ctrl (+FSK).

We have previously established an in vitro cyst model by the use of a subclone of Madin–Darby canine kidney cells which highly resemble principal cells of the collecting duct (principal-like (pl) MDCK cells)—the main origin of cyst development in ADPKD [33, 34]. plMDCK cells form cysts within a collagen I matrix and grow in a secretion-dependent manner sharing many characteristics with ADPKD cyst-lining cells [33, 35, 36]. plMDCK cells were either exposed to hypoxia (5% O_2_) or ICA (10 μM) for 24 h to stabilize HIF. We then tested plMDCK cells for MIF protein expression by the use of western blot and could detect a significant upregulation of MIF upon hypoxia and ICA (Fig. 3a and b). Since cyst growth in this model depends on cAMP according to the situation in vivo [4, 33, 37], we next treated the cells additionally with forskolin (FSK; 10 μM) for 24 h to test for cAMP-dependent effects on MIF expression. Forskolin resulted in a significant increase of MIF protein expression which could be additionally augmented by ICA (Fig. 3c, d) suggesting both, HIF-1α and cAMP as regulators of MIF.

### MIF inhibitor ISO-1 inhibits cyst growth in a dose-dependent manner

**Fig. 4:**
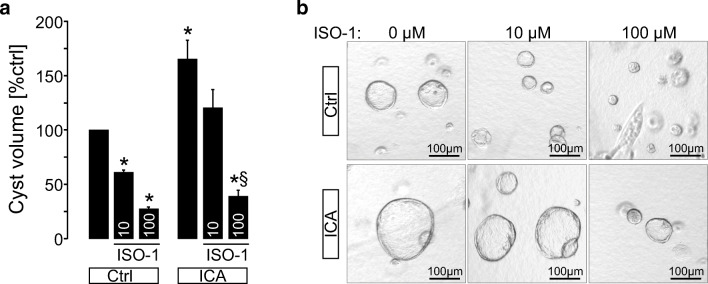
Inhibition of MIF reduces *in vitro* cyst growth in a dose-dependent manner and prevents ICA-dependent cyst enlargement. **a** plMDCK cells forming cysts within a collagen I matrix were exposed to control medium containing forskolin (10 μM) ± application of the MIF-inhibitor ISO-1 (10 μM and 100 μM) and ± application of ICA (10 μM). Graph shows mean cyst sizes after 5 days of treatment from three individual experiments comprising the analysis of 210–240 cysts per condition (control (absence of ISO-1) volume was set = 100%). **b** Representative cysts within the collagen matrix at day 5. *Significant compared with Ctrl. §Significant compared with ICA

In order to test if MIF may directly affect cyst growth (independent of macrophage recruitment), we tested for effects of recombinant MIF-protein (rMIF) at concentrations of 10 ng/ml and 100 ng/ml in our plMDCK cyst model but we did not find any significant effect on in vitro cyst growth (not shown). Of note, rMIF can only be applied from the basolateral site in the 3D cyst model. Next, we tested the cell permeable MIF-inhibitor 4,5-dihydro-3-(4-hydroxyphenyl)-5-isoxazoleacetic acid methyl ester (ISO-1) in our plMDCK cyst model. ISO-1 significantly inhibited cAMP-dependent cyst enlargement in a dose-dependent manner (Fig. 4). ICA resulted in an increase of cyst growth as shown previously [14, 15] which could also be attenuated by ISO-1 in a dose-dependent manner (Fig. 4). These data indicate, that inhibition of MIF reduces cAMP- and HIF-1α-dependent in vitro cyst enlargement.

### MIF-inhibitor ISO-1 inhibits and rMIF increases plMDCK cell proliferation

**Fig. 5:**
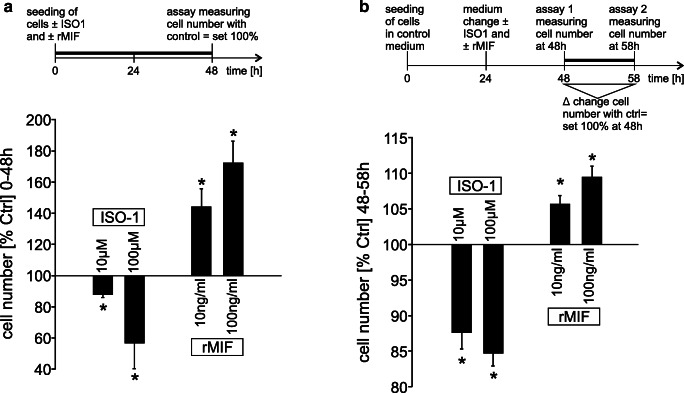
MIF promotes plMDCK cell proliferation. **a** plMDCK cells were seeded in 96 wells and grown in the presence and absence of rMIF (10 and 100 ng/ml) and ISO-1 (10 and 100 μM) for 48 h. Thereafter, a MTS assay was performed. Graph shows means of the obtained luminescence which correlates with the number of viable cells from *n* = 6 individual experiments and was normalized to control treated cells set = 100%. **b** plMDCK cells were seeded in 96 wells and grown in the presence of control medium for 24 h. Then, medium was changed and supplemented with the cell viability substrate, NanoLuc Enzyme and treated with ISO-1 (10 and 100 μM) or rMIF (10 and 100 ng/ml) for 24 h. Thereafter, the increase in luminescence from time point 48 to 58 h was measured. Graph shows the deviation of luminescence signal compared to control cells set = 100% in *n* = 6 individual experiments. *Significant compared with control

Next, we wanted to test if ISO-1-dependent decrease of cyst growth can be referred to reduction in cell proliferation. In addition, we wondered if apical application of rMIF (at the site of secretion in vivo) may affect cyst cell proliferation whereas basal application as done in the in vitro cyst assays may be ineffective. Therefore, MTS assays were performed in plMDCK cells grown in the presence and absence of rMIF and ISO-1 for 48 h showing significant reduction of cell number in the presence of ISO-1 and significantly increased cell number in the presence of rMIF (Fig. 5a). In order to verify these results and to exclude artifacts caused by potential differences in initial cell adhesion after seeding of the cells, we used another cell proliferation assay, and all cells were grown in the same control medium for 24 h. Then medium was changed, and cells were treated with ISO-1 or rMIF for 24 h. Thereafter, the increase of cell number from time point 48 to 58 h was measured at the different conditions. In concordance with the results above, ISO-1 reduced, whereas rMIF increased cell numbers (Fig. 5b). These data suggest that MIF promotes plMDCK cell proliferation.

### ISO-1 leads to a reduction of the cell proliferation marker proliferating cell nuclear antigen in plMDCK cysts

**Fig. 6:**
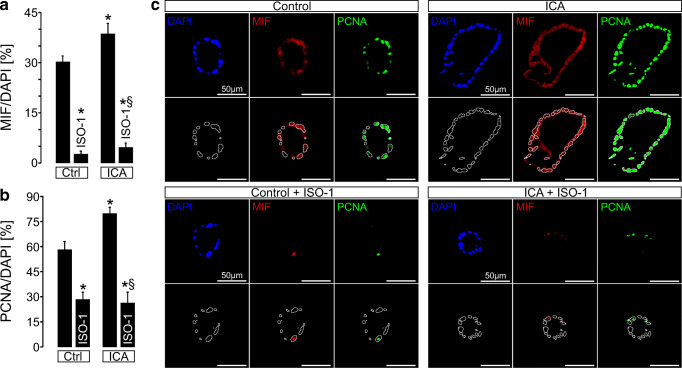
Inhibition of MIF results in reduction of in vitro cyst cell proliferation. plMDCK cells forming cysts were exposed to control medium containing forskolin (10 μM) ± application of the MIF-inhibitor ISO-1 (10 μM) and ± application of ICA (10 μM) for 5 days. **a** Quantification of MIF-positive cells in the cysts normalized to total cyst cell number obtained by DAPI staining. **b** Quantification of PCNA-positive cells in the cysts normalized to total cyst cell number obtained by DAPI staining. **a**, **b** Means of three individual experiments comprising the analysis of 120–130 cysts per condition. **c** Representative stainings of cysts for nuclei (DAPI; blue), MIF (red), and PCNA (green). Outline of nuclei stained by DAPI were marked by a white line to better visualize the proportion of PCNA- and MIF-positive cells and total cell number by overlay (lower rows). *Significant compared with Ctrl. §Significant compared with ICA

Since MIF promoted plMDCK cell proliferation in vitro, we next tested for proliferation markers in our plMDCK cyst model. Therefore, we stained the cysts for MIF and the cell proliferation marker proliferating cell nuclear antigen (PCNA) and found a correlation of both. Application of ICA was associated with an increase in MIF expression which was associated with an increase in PCNA (Fig. 6). In contrast, ISO-1 significantly reduced both, expression of MIF and PCNA (Fig. 6), suggesting that MIF-dependent cyst enlargement is due to increased cyst cell proliferation. Next to proliferation, apoptosis has also been shown to affect cyst growth [38]. Therefore, we performed TUNEL assays in our cyst model and found a reduction of apoptosis by application of ISO-1, whereas ICA led to an increased apoptosis rate (Supplemental Figure 8). These data indicate that MIF may affect apoptosis of cyst epithelial cells.

## Discussion

Continuous cyst expansion and subsequent compression of primarily unaffected kidney tissue is considered as the main driving force for the decline in renal function in ADPKD [2]. Therefore, identifying mechanisms of cyst growth is crucial for developing strategies to retard cyst growth and preserve renal function. Cyst growth is supposed to be mediated by at least three main forces: cyst cell proliferation, influx of fluid, and inflammation [1]. Clinical studies have been conducted targeting cyst cell proliferation by inhibition of mTOR or transepithelial cyst secretion by pharmacological inhibition of the vasopressin 2 receptor (V2R) [39–41]. These strategies were either not successful or showed quantitatively modest effects with relevant side effects [40, 41]. Thus, unravelling relevant pathways contributing to cyst growth in order to find druggable targets is of significant clinical interest.

We have previously shown that cyst growth is accompanied by regional hypoxia, which induces HIF-1α in cyst-lining cells which then promotes cyst growth primarily through induction of genes that mediate calcium-activated chloride secretion [13–15]. However, we also found HIF-1α-dependent increase of cyst cell proliferation in vivo, which was especially pronounced in a rapid progressive ADPKD mouse model [15]. The mechanisms, however, remained elusive. MIF has previously been described to be transcriptionally regulated by HIF-1α in cells of the trachea, lung and in cancer cells [22, 24, 26, 42, 43]. In addition, MIF has been shown to be upregulated and promote cyst growth in an ADPKD mouse model [9]. Our data demonstrate that MIF upregulation can be explained to some extent by HIF-1α-dependent transcriptional regulation in vivo and in vitro. The ChIP-seq signals from primary tubular cells derived in our study precisely overlap with the previously postulated HRE within the 5’ UTR of the MIF gene [24]. We therefore conclude that the functional element mediating HIF-binding in tubular cells is identical to the previously described one. Next to HIF-1α, we also found cAMP which is a main driver of cyst growth in ADPKD as an additional regulator of MIF expression. In line with these findings, MIF has recently been reported to be transcriptionally regulated by cAMP response element-binding protein (CREB) in immune and epithelial cells [44]. However, if cAMP-dependent upregulation of MIF can be referred to CREB in cyst epithelial cells has to be further analyzed in the future. Interestingly, HIF-1α and cAMP showed synergistic upregulation of MIF in cyst-forming cells. If this can be referred to different mechanisms of MIF regulation or if HIF-1α and cAMP merge into, a common pathway remains elusive at the moment.

HIF-1α stabilization by the prolyl-hydroxylase inhibitor ICA did not only result in an increase of MIF protein in cyst-lining cells but also induced cyst growth which could be inhibited by the MIF-inhibitor ISO-1. Therefore, these findings indicate that HIF-1α-mediated cyst cell proliferation could be in parts mediated by MIF. In addition, we also found a reduction in apoptosis in plMDCK cysts upon application of ISO-1 which is in line with a recent report that showed that mice lacking MIF had less apoptosis in acute kidney injury upon application of cisplatin [45]. In PKD, the role of apoptosis is still a matter of debate. While inhibition of apoptosis has been shown to delay renal cyst growth in some animal models of PKD, induction of apoptosis of cyst-lining epithelial cells has been shown to slow disease progression in Pkd1 knockout mice [46]. However, our results are in conflict with data showing an induction of apoptosis upon treatment with ISO-1 in an ADPKD mouse model [9]. Therefore, the exact role of MIF for apoptosis, and also potentially autophagy, and its contribution to cyst growth in ADPKD needs further investigation. Interestingly, although ICA also results in increased expression of MIF in wildtype kidneys, it does not result in cyst formation [47] (and Supplemental Figure 2). This confirms previous findings that HIF is not involved in early cyst formation but rather promotes cyst enlargement in advanced stages of PKD [15, 30].

Since MIF has been shown to be secreted [9], we tested for HIF-dependent regulation of ABCA1, a putative transporter protein of MIF [28]. In line with the hypothesis, that hypoxia triggers both, MIF expression and the induction of the MIF transporter ABCA1; we found that expression of ABCA1 is directly transactivated by HIF-1α. Therefore, HIF-1α could not only increase MIF expression but also directly affect MIF secretion via increased levels of ABCA1. However, although highly intuitive, further analyses will be needed to reliably confirm the transport function of ABCA1 for MIF in renal cells.

MIF has been shown to recruit macrophages by induction of monocyte chemotactic protein 1 (MCP-1) and induce proinflammatory pathways through, amongst others, tumor necrosis factor (TNF)-α. TNF-α may also promote cyst cell proliferation [48, 49]. Therefore, MIF-dependent cyst enlargement may either be driven indirectly via attracted macrophages or in a direct way. MIF may be capable of inducing cell proliferation by its increase in the cytosol as well as by extracellular application. Here, we show that extracellular application of rMIF increased cell proliferation. However, whether intracellular or extracellular expression of MIF is the dominant driver for cell proliferation (in the absence of macrophages) has to be further evaluated. Nevertheless, our data from the in vitro cyst model suggest that MIF mediates cyst growth not only by recruitment of macrophages but also in a direct manner. rMIF promoted cell proliferation in 2D cell cultures but did not affect in vitro 3D cyst growth. This may be explained by apical secretion of MIF into the cysts’ lumen [9] which suggests that MIF most likely acts from the cell’s apical site. This is in line with the observed increase of the MIF receptor CD74 and an indicated apical staining of CD74 in ADPKD cysts [9]. In the cyst model, however, rMIF can only be applied from the basolateral site. In contrast, ISO-1 is a cell-permeable isoxazoline compound, which therefore can act from both sites.

MIF is regulated by HIF-1α as well as cAMP, both of which have been shown to promote cyst enlargement in PKD. In addition, MIF secretion may also be affected by HIF-1α since the MIF transporter ABCA1 is also transcriptionally regulated by HIF-1α. Therefore, MIF could qualify as a druggable target downstream of HIF-1α and cAMP in order to reduce cyst enlargement and preserve renal function.

## Methods

### plMDCK cyst model

In vitro cyst assays were performed as described previously [50]. Principal-like Madin–Darby canine kidney (plMDCK) cells initially described as C7 cell clones by Gekle et al. [51] were a kind gift of Hans Oberleithner, University of Muenster as described previously [33]. plMDCK cells were resuspended as single-cell suspensions in a type I collagen matrix in 24-well plates (3–6 wells per condition) in presence of 37 °C and 21% O_2_/5% CO_2_ in modified MEM containing Earl’s balanced salt solution supplemented with 2 mM l-glutamine, 10% heat-inactivated FCS, 50 IU/ml penicillin, and 50 μg/ml streptomycin. Forskolin (Sigma-Aldrich), 2-(1-chloro-4-hydroxyisoquinoline-3-carboxamido) acetate (ICA) [29], and ISO-1 (Calbiochem, San Diego, USA) were added to the medium at day 0 and changed every 2 days. Experimental conditions were performed in the presence of forskolin. After 5 days, four random visual fields per well were photographed with a Zeiss Primo Vert microscope and a Zeiss Axiocam 105 color camera (both Zeiss Microscopy GmbH, Jena, Germany). Cyst diameters of all captured cysts that were nearly spherical were measured with ImageJ (V. 1.48) and the use of a Wacom Tablet device. Cyst volume was then estimated using the formula for the volume of a sphere, 4/3*πr*^3^.

### ChIP-seq data

Data from HIF ChIP-seq experiments in human primary tubular cells were generated in previous work (GEO data set: GSE101064, sample GSM2698833 for HIF-1α, and sample GSM2698834 for HIF-1β) [32, 52] and visualized using the USCS Genome browser (https://genome.ucsc.edu/). For chromatin immunoprecipitation, we used HIF-1α antibodies (6 μl; Cay10006421, Cayman Chemicals, Ann Arbor, MI) and HIF-1β antibodies (6 μl; NB100-110, Novus Biologicals).

### Primary renal tubular cells

Healthy human kidney cortical tissue from patients undergoing tumor nephrectomy was used for isolation of tubular cells. Informed consent was given by each patient, and the use of the tissue was approved by the local ethical committee at the University of Erlangen-Nuernberg (Approval numbers: 3755 and 173_15Bc). Cells were isolated, cultured and exposed to 1 mM dimethyloxalylglycine (DMOG, Cayman Chemical, Ann Arbor, MI, USA) for 16 h to stabilize HIF according to the protocol published by Grampp et al. [32].

### siRNA transfection and RNA quantification

siRNA targeting HIF-1α was conducted as described previously [53]; siRNA against HIF-1β was purchased from Dharmacon (#L-007207-00-0005; Lafayette, CO, USA). For primer sequences, see Supplementary Table 1. Transfection and qPCR were performed as described earlier [32]. Briefly, siRNAs at a final concentration of 40 nM were transfected using Saint red (Synvolux, Leiden, The Netherlands) transfection reagent. Transfection was repeated after 24 h, and cells were harvested 48 h after the initial transfection. RNA from cells or tissue was isolated using Tri Reagent (Sigma-Aldrich) or peqGold total RNA kit (PeqLab, Erlangen, Germany) according to the manufacturer’s protocol and transcribed into cDNA using the high capacity cDNA reverse transcription kit (Life Technologies, Carlsbad, CA, USA). qPCRs were performed on a StepOnePlus real-time PCR system (Applied Biosystems, Foster City, CA, USA).

### Animal experiments

All animal experiments were ethically approved by local government authorities and conformed to the US National Institutes of Health Guide for the Care and Use of Laboratory Animals. In this study, kidney sections of a tamoxifen-inducible kidney epithelium-specific Pkd1 deletion mouse model crossed with a HIF-1α knock-out murine model were used. Generation of the tamoxifen-inducible, kidney epithelium–specific *Pkd1*-deletion mouse model carrying the loxP-flanked conditional alleles of *Pkd1* (Ksp*CreER*^T2^;*Pkd1*^lox;lox^) has been described previously [54]. Upon administration of tamoxifen to the mice, a genomic fragment containing exons 2–11 of the Pkd1 gene is specifically deleted in renal epithelial cells inducing cyst formation [54]. Ksp*CreER*^T2^;*Pkd1*^lox;lox^ mice were crossed with mice carrying loxP-flanked alleles of HIF-1α (*Hif-1α*^fl;fl^) as described previously in order to receive tamoxifen-inducible, kidney epithelium–specific deletion of *Pkd1* and *Hif-1α* (Ksp*CreER*^T2^*Pkd1*^lox;lox^;*Hif-1α*^lox;lox^) [15]. Genotyping was carried out by PCR on tail DNA samples. For genotyping primers, see Supplementary Table 2. Gene deletion was induced by daily intraperitoneal administration of tamoxifen (2 mg/kg body weight) dissolved in 5% ethanol and 95% neutral oil from postnatal days (PN) 35–37 to induce a slow disease progression in male mice. Ksp*CreER*^T2^;*Pkd1*^lox;lox^ mice that received vehicle only (without tamoxifen) at the same time points served as controls. PN 35–37 induced Ksp*CreER*^T2^;*Pkd1*^lox;lox^ mice (males, *n* = 7;) and Ksp*CreER*^T2^;*Pkd1*^lox;lox^*;Hif-1α*^lox;lox^ mice (male: *n* = 5) received ICA by intraperitoneal injection (40 mg/kg body weight; dissolved in 5% DMSO and 95% 0.5 M Tris) 5 days per week for 12 weeks and were compared with Ksp*CreER*^T2^;*Pkd1*^lox;lox^ mice (males, n = 6) and Ksp*CreER*^T2^;*Pkd1*^lox;lox^;*Hif-1α*^lox;lox^ mice (males, *n* = 6) that received vehicle (with no ICA). Noninduced but vehicle-treated Ksp*CreER*^T2^; *Pkd1*^lox;lox^ mice (males, *n* = 4) served as controls. All PN 35–37–induced animals were observed for 12 weeks. Thereafter, animals were sacrificed and kidneys were harvested. The mice of which samples were utilized for this study were characterized for their cystic index as well as serum creatinine levels in our previous paper [15].

### Immunoblotting

plMDCK cells were exposed to control medium, medium supplemented with either forskolin (FSK; 10 μM) or ICA (10 μM), or the combination of both, or exposed to 5% O_2_ for 24 h. Proteins were dissolved in lysis buffer containing 6.65 M urea, 10% glycerol, 10 mM Tris-HCl (pH 6.8), 1% SDS, 5 mM dithiothreitol, and complete protease inhibitors (Roche) and sonicated. Fifty micrograms of proteins were used for immunodetection of MIF. Proteins were detected with the primary polyclonal rabbit anti MIF antibody (1:1000; Sigma-Aldrich, Missouri, USA). As secondary antibody, a polyclonal goat antirabbit (1:10000, Dako, Hamburg, Germany) antibody was used. For detection of HIF-1α and HIF-1β, 50 μg of primary renal tubular cells were used. Proteins were detected with the primary polyclonal rabbit antiHIF-1α antibody (1:1000; Cay10006421, Cayman Chemicals, Ann Arbor, MI) and polyclonal rabbit antiHIF-1β antibody (1:1000; NB100-110, Novus Biologicals). As secondary antibodies, an antirabbit IgG (1:500; Vector, Burlingame, CA) was used. Beta actin was detected with a HRP-coupled rabbit antibody (1:30000, Sigma Aldrich).

### Immunofluorescence, immunohistochemistry, and antibodies

plMDCK cysts at day 5 were rinsed in phosphate-buffered saline (PBS) supplemented with 0.9 mmol/l calcium chloride and 0.49 mmol/l magnesium chloride (PBS+). Paraformalde-hyde (4%) was added to fix the cysts for 1 h at room temperature. Glycine (200 mmol/l) in PBS+ was added for another hour to quench the excess aldehyde. Three-micrometer paraffin sections were costained for MIF and PCNA. Sections were boiled for 7 min in a steam pressure pot (120 °C) in a modified citrate buffer (Target Retrieval Solution; S1699, Agilent, Waldbronn, Germany). Blocking was performed using 1% BSA/PBS^+^ for 30 min. at room temperature. Primary rabbit MIF- (FI-115, 1:100, Santa Cruz, Heidelberg, Germany) and mouse PCNA antibody (M0879, 1:100, Dako) were incubated overnight. After washing with PBS^+^, the binding of the primary antibodies were visualized using secondary rabbit Alexa Fluor 555 and mouse Alexa Fluor 488 (1:1000, Thermo Fisher Scientific, Inc., Erlangen, Germany) for 90 min. For detection of MIF in the kidney, three-micron transverse kidney sections, fixed overnight in paraformaldehyde (4%) and embedded in paraffin, were stained. Sections were deparaffinized and were boiled at 120 °C in a steam pressure pot for 6.5 min. Sections were exposed to 3% peroxidase for 10 min before incubation in horse serum (ImmPRESS horse antirabbit IgG Polymer kit, Vector, Burlingame, CA) for 20 min. Primary rabbit MIF antibody (FI-115, 1:100) was incubated at 4 °C overnight. Thereafter, the sections were washed in Tris-buffered saline with Tween20 (TBST) and treated with rabbit ImPess Kit (30 min, MP-7401, Vector). Visualization of the MIF signal was performed using Diaminobenzidin (K1500 from CSA kit, Dako). For detection of ABCA1, kidney sections were boiled in a steam pressure pot at 120 °C in Target Retrieval Solution for 5 min. After washing in TBST the sections were blocked using AVIDIN/BIOTIN Blocking kit (P2001, Vector, Burlingame, CA, USA) for 20 min. before adding 3% peroxidase for 10 min. Sections were incubated in protein block serum free (X0909, Dako) for 60 min. Primary antibody rabbit IgG polyclonal ABCA1 (1:500, Thermo Fisher Scientific, Inc.) was incubated at 4 °C overnight. After washing with TBST the ABCA1 signal was detected using a secondary goat biotinylated IgG antibody (1:500, Vector) for 30 min. at room temperature. Thereafter, a Vectastain ABC-Komplex (Vector PK-6100, 15 min.) was added. In a further step Biotin Plus Amplification Reagent TSA Plus Biotin Kit (1:50, PerkinElmer, Hamburg, Germany) for 15 min. was added. Finally, an incubation step in Streptavidin (Dako) was added for 30 min. Visual detection of the signal was performed using a chromogen detection solution Diaminobenzidin (K1500 from CSA kit, Dako) plus substrate buffer concentrate (K1500 from CSA kit, Dako). A hematoxylin by Gill (Merck, Darmstadt, Germany) counterstaining was performed in addition. For detection of HIF-1α, polyclonal rabbit antiHIF-1α (1:5000; Cayman Chemicals, Ann Arbor, MI) primary antibody was used. Thereafter, a catalyzed signal amplification system and a biotinylated secondary antirabbit IgG (1:500; Vector, Burlingame, CA) were used. Dolichos biflorus agglutinin (DBA) was visualized using fluorescein-labeled DBA (Vector Laboratories) to identify collecting ducts. Proximal tubules were detected by their characteristic brush borders and green autofluorescence. Signals were captured with a DM6000B fluorescence microscope (Leica, Wetzlar, Germany), and photographs were taken with a Leica DFC 450C camera.

### Quantification of MIF and ABCA1 staining

Eight random photographs were taken from the cortex and medulla of each kidney (Ctrl: *n* = 5; *Pkd1*^fl;fl^: *n* = 6; *Pkd1*^fl;fl^*;Hif-1α*^fl;fl^: *n* = 6; *Pkd1*^fl;fl^ + ICA: *n* = 7; *Pkd1*^fl;fl^;*Hif-1α*^fl;fl^ + ICA: *n* = 5) at a magnification of × 200. Immunohistochemical signals of MIF and ABCA1 were analyzed as described previously [15]. Briefly, a color deconvolution algorithm (ImageJ software version 1.48) was applied to dissect the different signals and turned into 8-bit Lookup Table (LUT) images, followed by binarization and particle analysis to obtain the ratio of positive area per total cortex or medullary area. Fluorescent signals were analyzed and normalized as described previously [55].

### Analysis of subcellular localization of MIF

A total of *n* = 337 tubular segments or cysts from *n* = 3 individual Ksp*CreER*^T2^;*Pkd1*^lox;lox^ mouse kidneys stained for MIF were separated into normal tubules defined by a luminal diameter < 50 μm, dilated tubules defined by diameters between 50 and 100 μm and cysts defined by diameters > 100 μm by the use of ImageJ (V. 1.48) and analyzed for either cytoplasmic MIF staining patterns (no signal in nucleus) or nuclear staining patterns (apparent nuclear signal).

### Cell proliferation assays

Two assays were performed to analyze cell proliferation. First, a CellTiter 96 Aqueous One Solution Cell Proliferation Assay (G3582, Promega), also known as classical MTS assay, was performed according to the manufacturer’s protocol. Cells were seeded (1250 cells/well) in a 96-well plate in the presence or absence of 10 and 100 μM ISO-1 or 10 and 100 ng/ml recombinant MIF (rMIF), respectively. After 47 hours, CellTiter 96 Aqueous One Solution Reagent was added to each well and absorbance was measured after 1 h of incubation time. Second, the RealTime-Glo MT Cell Viablility Assay (G9711, Promega) was performed according to the manufacturer’s protocol. Cells were seeded into 96-well plates (1250 cells/well) in control medium. Twenty-four hours later, medium was changed and supplemented with Cell Viability Substrate, NanoLuc Enzyme, and 0, 10, and 100 μM ISO-1 or 0, 10, and 100 ng/ml recombinant MIF (rMIF), respectively. Twenty-four hours later, luminescence was measured with an integration time of 0.5 s which served as starting point. Ten hours later, luminescence was measured again, and the delta increase in the obtained values was analyzed.

### TUNEL assay

Terminal deoxynucleotidyl transferase dUTP nick end labeling (TUNEL) Assays were performed utilizing the Molecular Probes Click-iT Plus TUNEL Assay (C10618, Invitrogen). Paraffin sections of plMDCK cysts were stained according to the manufacturer’s protocol.

### Quantification of TUNEL- and PCNA signals in plMDCK cysts

plMDCK cysts were stained for DAPI, MIF, PCNA, and TUNEL after 5 days of application of control medium containing forskolin (10 μM) and supplemented with the MIF-inhibitor ISO-1 (10 μM) or ICA (10 μM). *n* = 120–130 cysts per condition from three individual experiments were analyzed for MIF, PCNA, and TUNEL-positive cells within the cysts and normalized to total cyst cell number obtained by DAPI staining. Outline of nuclei stained by DAPI were marked by a white line by ImageJ (V.1.48) to better visualize the fraction of PCNA-, MIF-, and TUNEL-positive cells.

### Statistical analysis

Data are expressed as mean ± SEM. Differences among groups were analyzed using one-way ANOVA, followed by a Bonferroni test for multiple comparisons. An unpaired *t* test was applied to compare the differences between two groups. Wilcoxon signed-rank test for columns statistics was used for relative values. *P* < 0.05 was considered statistically significant.

## Electronic supplementary material

Supplemental Figure 1 MIF and ABCA1 are expressed in a HIF-1α-dependent manner in cyst-lining cells of an ADPKD mouse model. Tamoxifen was applied at postnatal day 35-37 to induce tubule-specific deletion of PKD1 in Ksp*CreER*^T2^;*Pkd1*^lox;lox^ (*Pkd1*^fl;fl^; *n* = 7) mice. In parallel, genetic deletion was induced in Ksp*CreER*^T2^;*Pkd1*^lox;lox^;Hif-1α^lox/lox^ (*Pkd1*^fl;fl^;*Hif-1α*^fl;fl^; *n* = 5) mice to receive tubular codeletion of PKD1 and HIF-1α. Mice were then either treated with the prolylhydroxylase inhibitor 2-(1-chloro-4-hydroxyisoquinoline-3-carboxamido) acetate (*Pkd1*^fl;fl^ + ICA; *n* = 6); (*Pkd1*^fl;fl^;*Hif-1α*^fl;fl^ + ICA; *n* = 6) or its vehicle for 12 weeks. Noninduced mice served as controls (Ctrl; *n* = 4). **A** As shown previously, the abovementioned ADPKD mouse model (*Pkd1*^fl;fl^) shows a mild progression which does not lead to hypoxia or induction of HIF-1α. In line with these findings, MIF expression did not differ in the medulla between Ctrl, *Pkd1*^fl;fl^, and *Pkd1*^fl;fl^;*Hif-1α*^fl;fl^ kidneys. However, application of ICA (*Pkd1*^fl;fl^ + ICA) resulted in a significant increase of HIF-1α shown previously which was prevented in mice co-deleted for HIF-1α (*Pkd1*^fl;fl^;*Hif-1α*^fl;fl^ + ICA). In line with the assumption of MIF being regulated by HIF-1α, MIF expression was significantly increased in the medulla of *Pkd1*^fl;fl^ + ICA mice which could be prevented in mice co-deleted for HIF-1α (*Pkd1*^fl;fl^;*Hif-1α*^fl;fl^ + ICA). **B** ABCA1 shows a comparable pattern of expression to MIF in cyst cells in the medulla of the chosen models. *Significant compared with Ctrl. §Significant compared with *Pkd1*^fl;fl^ + ICA (PNG 122 kb)

High resolution image (TIF 17300 kb)

Supplemental Figure 2 HIF-induction results in increased expression of MIF and ABCA1 in wildtype mouse kidneys. Wildtype littermate mice were either treated with the prolylhydroxylase inhibitor 2-(1-chloro-4-hydroxyisoquinoline-3-carboxamido) acetate (Ctrl + ICA; *n* = 3) or its vehicle (Ctrl; *n* = 3) and sacrificed 24 h later. **A** Analysis of kidneys stained for MIF of Ctrl and ICA-treated mice. Right: Representative stainings for MIF (green), nuclei (blue). **B** Analysis of kidneys stained for ABCA1 of Ctrl and ICA-treated mice. Right: Representative stainings for ABCA1 (red), nuclei (blue). *Significant compared with Ctrl (PNG 1450 kb)

High resolution image (TIF 31516 kb)

Supplemental Figure 3 Subcellular localization of MIF depends on the degree of cyst formation. Tubules and cysts (*n* = 337) from n = 3 Ksp*CreER*^T2^;*Pkd1*^lox;lox^ mouse kidneys stained for MIF were classified into normal tubules (luminal diameter < 50 μm), dilated tubules (diameters between 50 and 100 μm) and cysts (diameters > 100 μm) and analyzed for either cytoplasmic MIF staining patterns (no signal in nucleus) or nuclear staining patterns (apparent nuclear signal). *Significant compared with “<50 μm”. §Significant compared with “50-100 μm” (PNG 94 kb)

High resolution image (TIF 24623 kb)

Supplemental Figure 4 MIF and ABCA1 are coexpressed in cyst-lining cells in vivo. Since ABCA1 has been shown to act as a transport protein for MIF, we stained serial sections of kidneys from Ksp*CreER*^T2^;*Pkd1*^lox;lox^ mice treated with ICA for ABCA1 or MIF, respectively, in order to test for co-expression of ABCA1 (red) and MIF (green). Large fields of view of kidney sections confirm distinct co-expression of both proteins. Areas within the white squares numbered from 1 to 4 were magnified to further illustrate ABCA1- and MIF coexpression within representative cyst-lining cells. (1) shows a normal tubule with nuclear MIF expression and coexpression of ABCA1. (2) shows small cysts with a more cytoplasmic expression of MIF and coexpression with ABCA1. (3) shows a large cyst with cytoplasmic MIF expression and ABCA1 coexpression. (4) shows one of the few MIF-negative cysts which does also not express ABCA1 (PNG 4714 kb)

High resolution image (TIF 80631 kb)

Supplemental Figure 5 The fraction of MIF- and ABCA1-positive cysts depends on HIF-1α in the ADPKD mouse model. In addition to the analyses in Fig. 1, the fraction of MIF- and ABCA1-positive cysts were analyzed in *Pkd1*^fl;fl^ (n = 1708 cysts from n = 6 mice), *Pkd1*^fl;fl^;*Hif-1α*^fl;fl^ (n = 1618 cysts from n = 6 mice), *Pkd1*^fl;fl^ + ICA (n = 1319 cysts from n = 6 mice) and *Pkd1*^fl;fl^;*Hif-1α*^fl;fl^ + ICA (n = 1469 cysts from n = 6 mice) mice. *Significant compared with *Pkd1*^fl;fl^. §Significant compared with *Pkd1*^fl;fl^ + ICA (PNG 117 kb)

High resolution image (TIF 25292 kb)

Supplemental Figure 6 MIF expression is not limited to specific tubular segments but correlates with expression of HIF-1α. **A** *Pkd1*^fl;fl^ kidneys were stained for MIF (green) and nuclei (DAPI; blue) and analyzed for segment-specific expression. Upper row, left: MIF can be found in collecting ducts stained by dolichos biflorus agglutinin (DBA; red). Right: Collecting ducts can also be negative for MIF. Lower row, left: Proximal tubules (marked by white lines) can be stained positive for MIF, but also negative (right). **B** Kidneys from *Pkd1*^fl;fl^ mice treated with ICA also show collecting ducts that are positive for MIF (upper row, left), but also a few that are negative (right). Lower row left shows proximal tubules that are stained positive for MIF. Right shows proximal tubule that is stained negative for MIF. **C** Kidneys from *Pkd1*^fl;fl^ mice treated with ICA were stained for MIF (green), nuclei (DAPI; blue) and HIF-1α (red). Left photos show cysts expressing HIF-1α and to a great extent co-expression of MIF in serial sections. Right shows HIF-1α-negative cysts and for the most part absence of MIF expression. **D** Kidneys from *Pkd1*^fl;fl^;*Hif-1α*^fl;fl^ mice treated with ICA were stained for MIF (green), nuclei (DAPI; blue) and HIF-1α (red). Left photos show typical cysts lacking HIF-1α and also MIF in serial sections. Right shows one of few HIF-1α-positive cysts and to a great extent coexpression of MIF (PNG 3252 kb)

High resolution image (TIF 64758 kb)

(PNG 2824 kb)

High resolution image (TIF 64206 kb)

Supplemental Figure 7 HIF protein is reduced in hPTCs by siRNA directed against HIF-1α or HIF-1β. Human primary renal tubular cells were either treated with siRNA directed against HIF-1α or HIF-1β, respectively and stimulated with DMOG for 16 hours. Representative western blot showing reduced levels of HIF-1α or HIF-1β on protein level upon application of siRNA directed against HIF-1α or HIF-1β, respectively (PNG 183 kb)

High resolution image (TIF 16359 kb)

Supplemental Figure 8 Inhibition of MIF results in reduction of in vitro cyst cell apoptosis. plMDCK cells forming cysts were exposed to control medium containing forskolin (10 μM) ± application of the MIF-inhibitor ISO-1 (10 μM) and ± application of ICA (10 μM) for 5 days. **A** Quantification of TUNEL-positive cells in the cysts (n = 120-130 cysts per condition) normalized to total cyst cell number obtained by DAPI staining. **B** Representative stainings of cysts for nuclei (DAPI; blue) and TUNEL (red). Outline of nuclei stained by DAPI were marked by a white line to better visualize the fraction of TUNEL-positive cells and total cell number by overlay (lower rows). *Significant compared with Ctrl. § significant compared to ICA (PNG 291 kb)

High resolution image (TIF 34995 kb)

ESM 1 (DOCX 13 kb)
