# Supplementary figures and images for: Macrophage migration inhibitory factor is regulated by HIF-1α and cAMP and promotes renal cyst cell proliferation in a macrophage-independent manner
Source: J Mol Med (Berl). 2020 Sep 4;98(11):1547–59. doi: 10.1007/s00109-020-01964-1 (PMC7591438; doi:10.1007/s00109-020-01964-1)

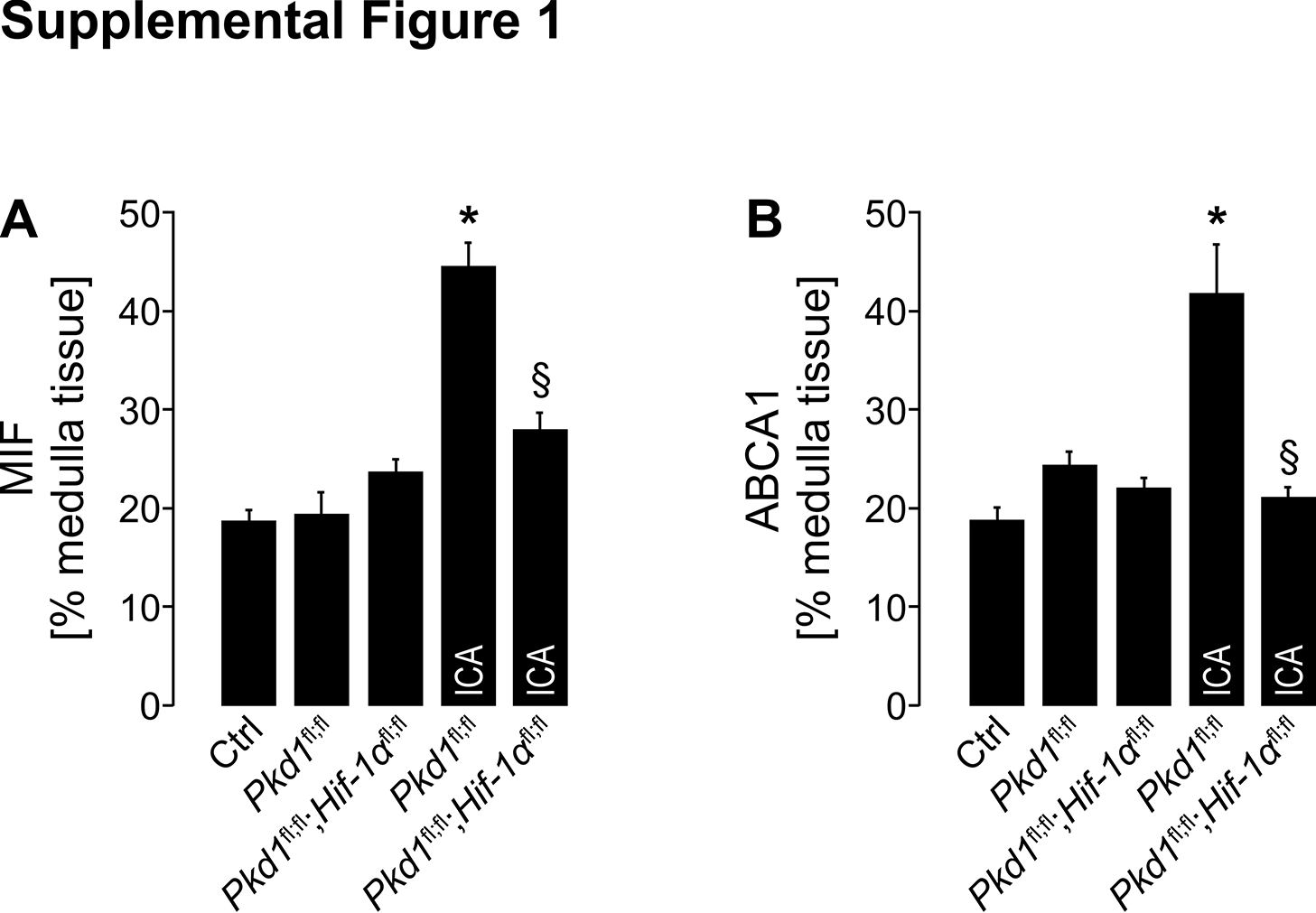

Supplement: Supplementary file 1 — MIF and ABCA1 are expressed in a HIF-1α-dependent manner in cyst-lining cells of an ADPKD mouse model. Tamoxifen was applied at postnatal day 35-37 to induce tubule-specific deletion of PKD1 in KspCreERT2;Pkd1lox;lox (Pkd1fl;fl; n = 7) mice. In parallel, genetic deletion was induced in KspCreERT2;Pkd1lox;lox;Hif-1αlox/lox (Pkd1fl;fl;Hif-1αfl;fl; n = 5) mice to receive tubular codeletion of PKD1 and HIF-1α. Mice were then either treated with the prolylhydroxylase inhibitor 2-(1-chloro-4-hydroxyisoquinoline-3-carboxamido) acetate (Pkd1fl;fl + ICA; n = 6); (Pkd1fl;fl;Hif-1αfl;fl + ICA; n = 6) or its vehicle for 12 weeks. Noninduced mice served as controls (Ctrl; n = 4). A As shown previously, the abovementioned ADPKD mouse model (Pkd1fl;fl) shows a mild progression which does not lead to hypoxia or induction of HIF-1α. In line with these findings, MIF expression did not differ in the medulla between Ctrl, Pkd1fl;fl, and Pkd1fl;fl;Hif-1αfl;fl kidneys. However, application of ICA (Pkd1fl;fl + ICA) resulted in a significant increase of HIF-1α shown previously which was prevented in mice co-deleted for HIF-1α (Pkd1fl;fl;Hif-1αfl;fl + ICA). In line with the assumption of MIF being regulated by HIF-1α, MIF expression was significantly increased in the medulla of Pkd1fl;fl + ICA mice which could be prevented in mice co-deleted for HIF-1α (Pkd1fl;fl;Hif-1αfl;fl + ICA). B ABCA1 shows a comparable pattern of expression to MIF in cyst cells in the medulla of the chosen models. *Significant compared with Ctrl. §Significant compared with Pkd1fl;fl + ICA (PNG 122 kb) [file 109_2020_1964_Fig7_ESM.png]

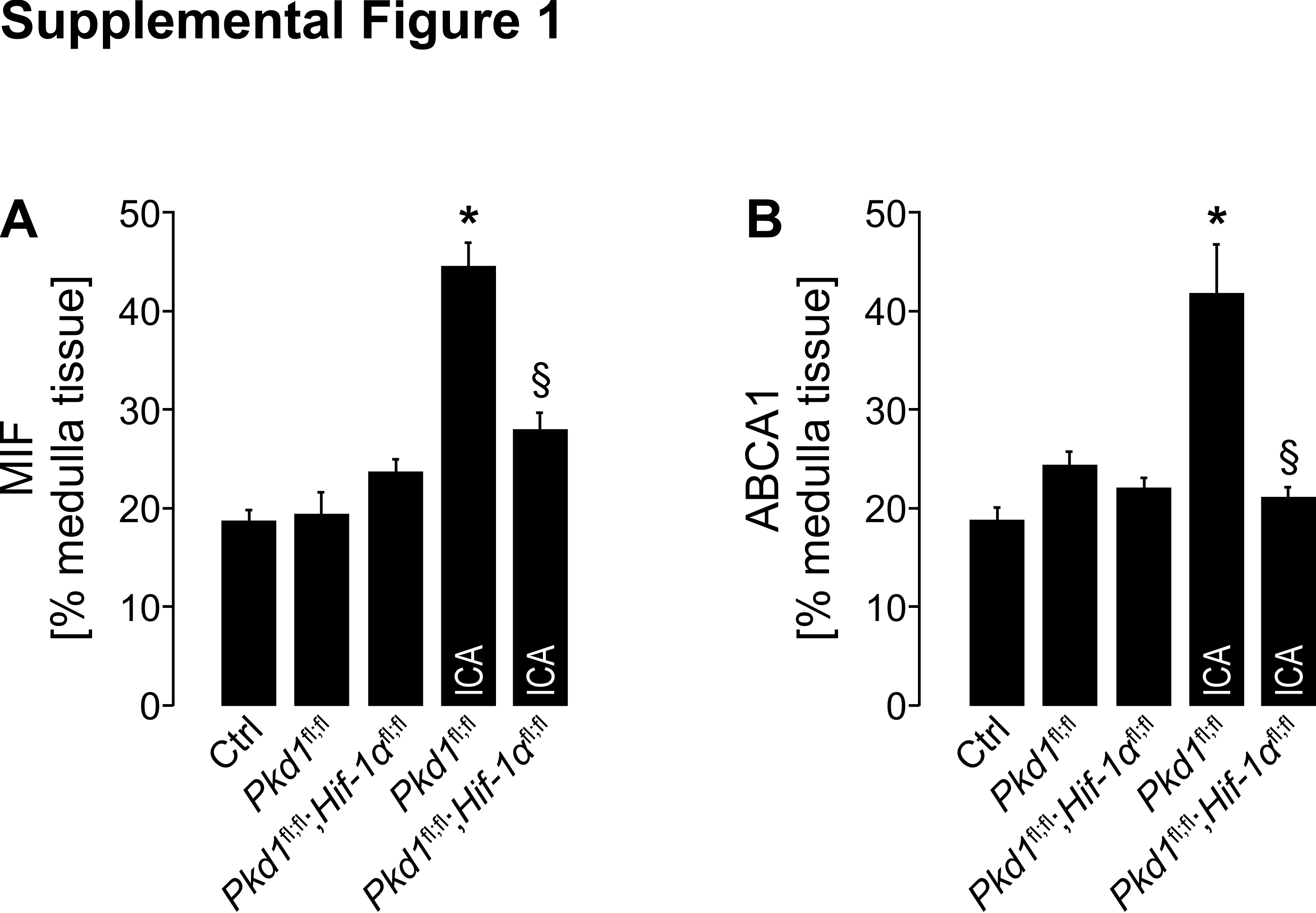

Supplement: Supplementary file 2 — High resolution image (TIF 17300 kb) [file 109_2020_1964_MOESM1_ESM.tif]

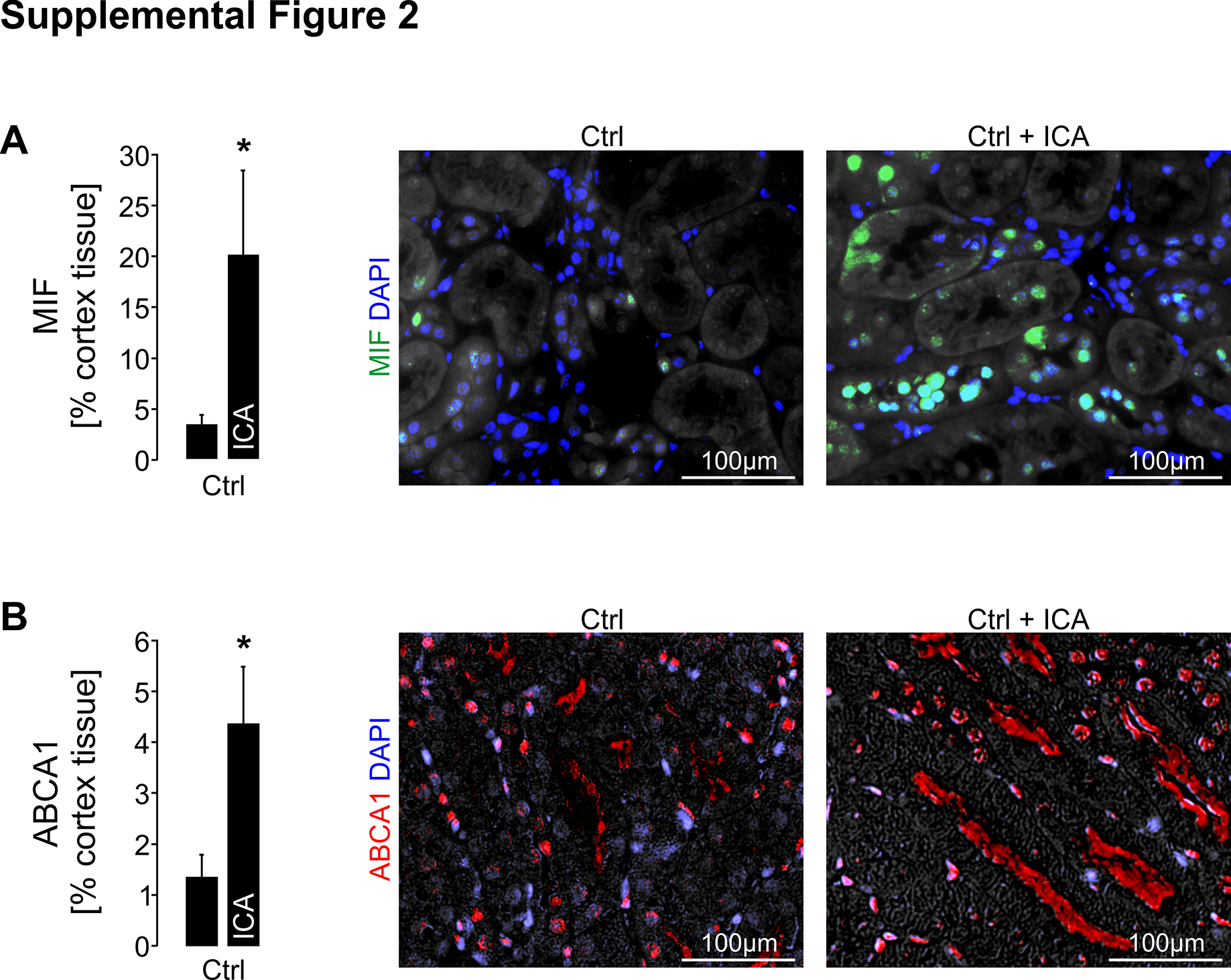

Supplement: Supplementary file 3 — HIF-induction results in increased expression of MIF and ABCA1 in wildtype mouse kidneys. Wildtype littermate mice were either treated with the prolylhydroxylase inhibitor 2-(1-chloro-4-hydroxyisoquinoline-3-carboxamido) acetate (Ctrl + ICA; n = 3) or its vehicle (Ctrl; n = 3) and sacrificed 24 h later. A Analysis of kidneys stained for MIF of Ctrl and ICA-treated mice. Right: Representative stainings for MIF (green), nuclei (blue). B Analysis of kidneys stained for ABCA1 of Ctrl and ICA-treated mice. Right: Representative stainings for ABCA1 (red), nuclei (blue). *Significant compared with Ctrl (PNG 1450 kb) [file 109_2020_1964_Fig8_ESM.png]

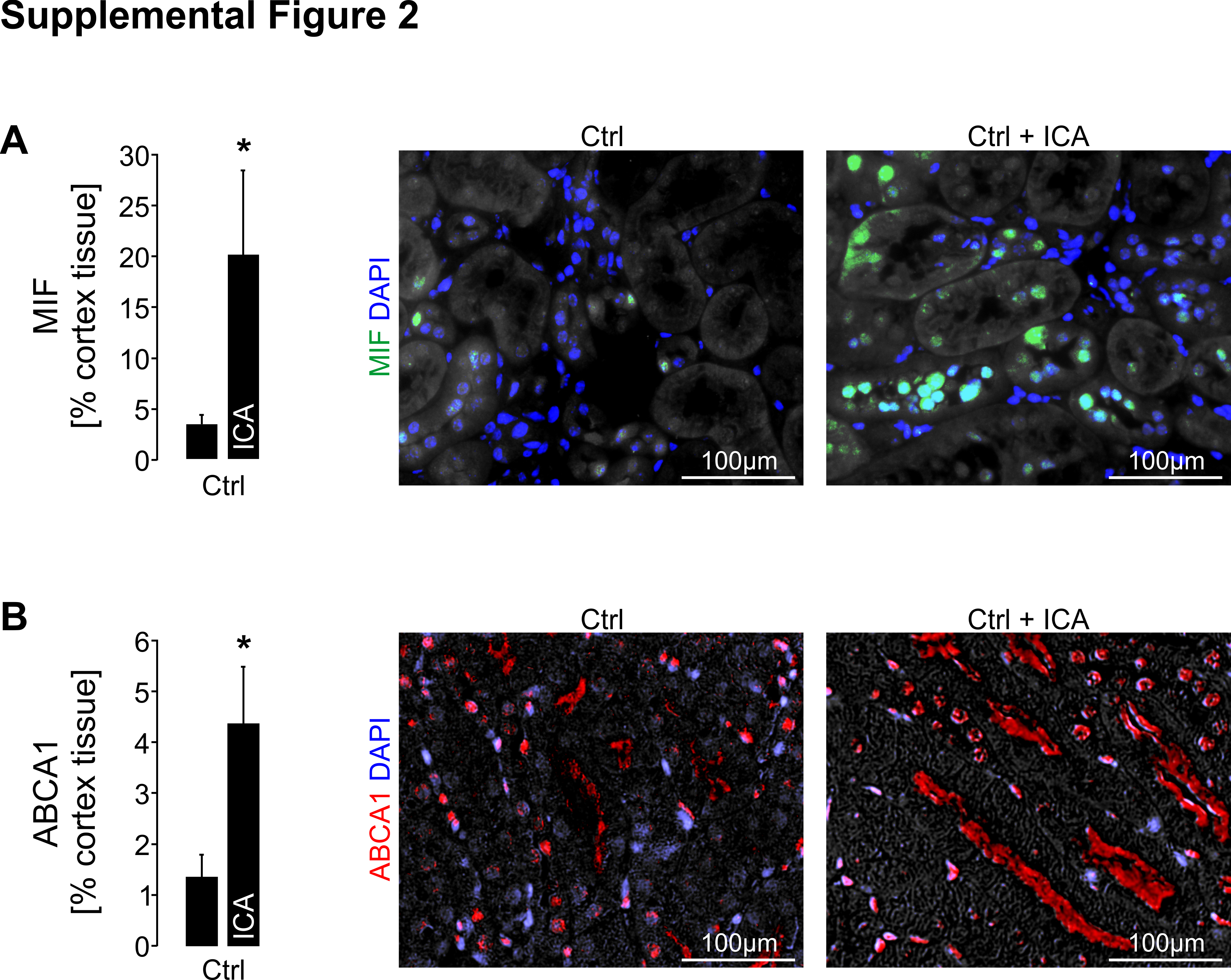

Supplement: Supplementary file 4 — High resolution image (TIF 31516 kb) [file 109_2020_1964_MOESM2_ESM.tif]

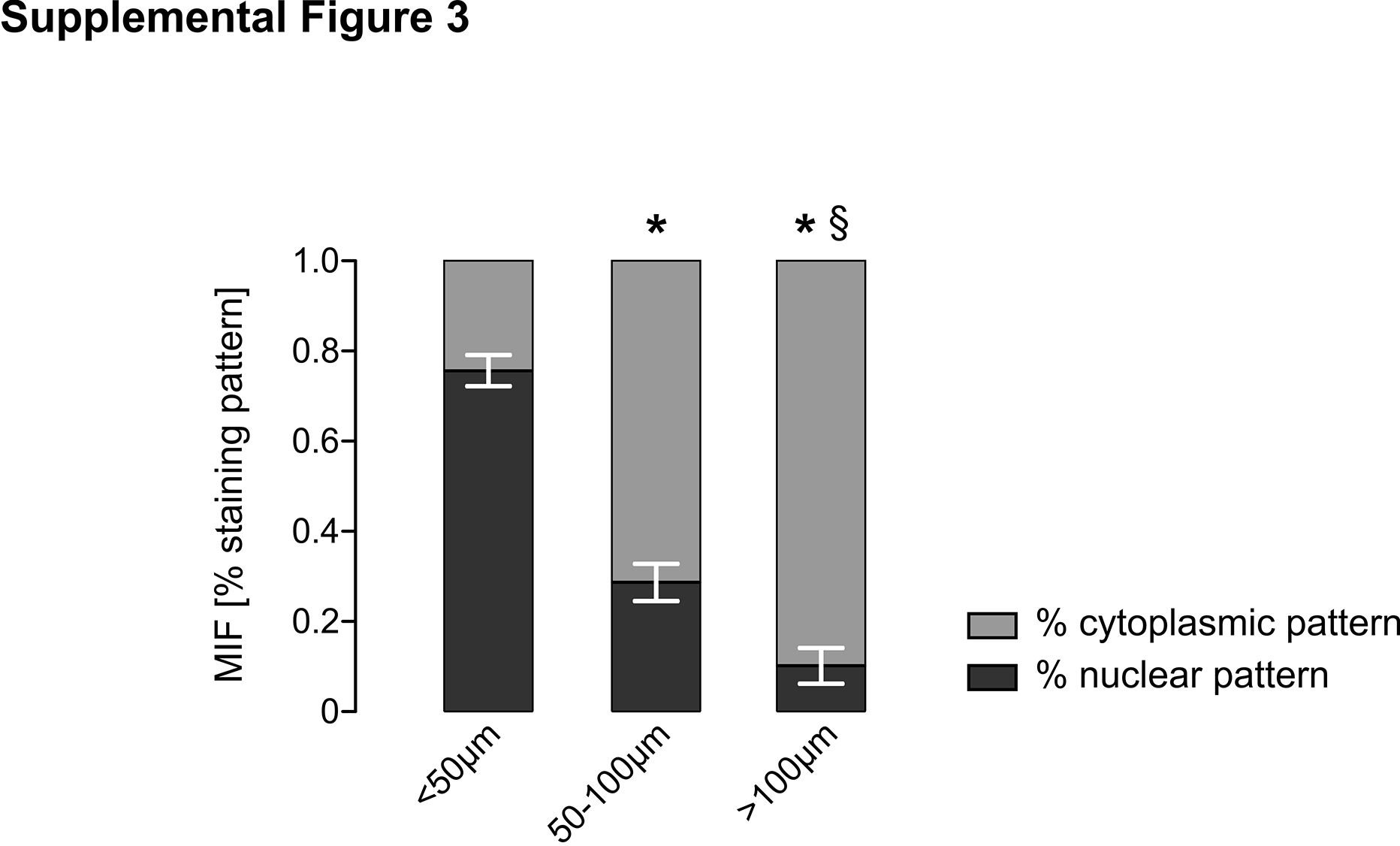

Supplement: Supplementary file 5 — Subcellular localization of MIF depends on the degree of cyst formation. Tubules and cysts (n = 337) from n = 3 KspCreERT2;Pkd1lox;lox mouse kidneys stained for MIF were classified into normal tubules (luminal diameter < 50 μm), dilated tubules (diameters between 50 and 100 μm) and cysts (diameters > 100 μm) and analyzed for either cytoplasmic MIF staining patterns (no signal in nucleus) or nuclear staining patterns (apparent nuclear signal). *Significant compared with “<50 μm”. §Significant compared with “50-100 μm” (PNG 94 kb) [file 109_2020_1964_Fig9_ESM.png]

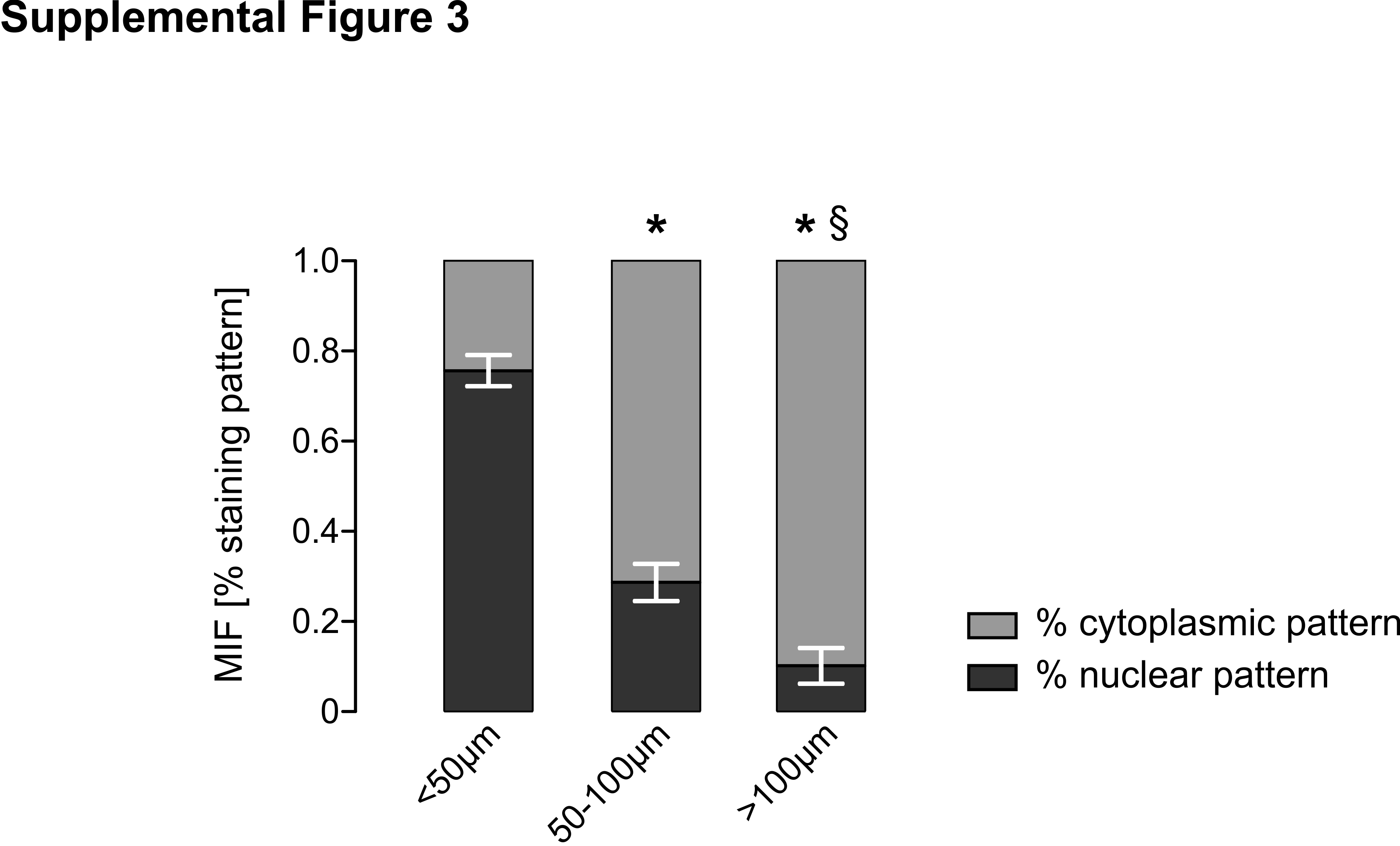

Supplement: Supplementary file 6 — High resolution image (TIF 24623 kb) [file 109_2020_1964_MOESM3_ESM.tif]

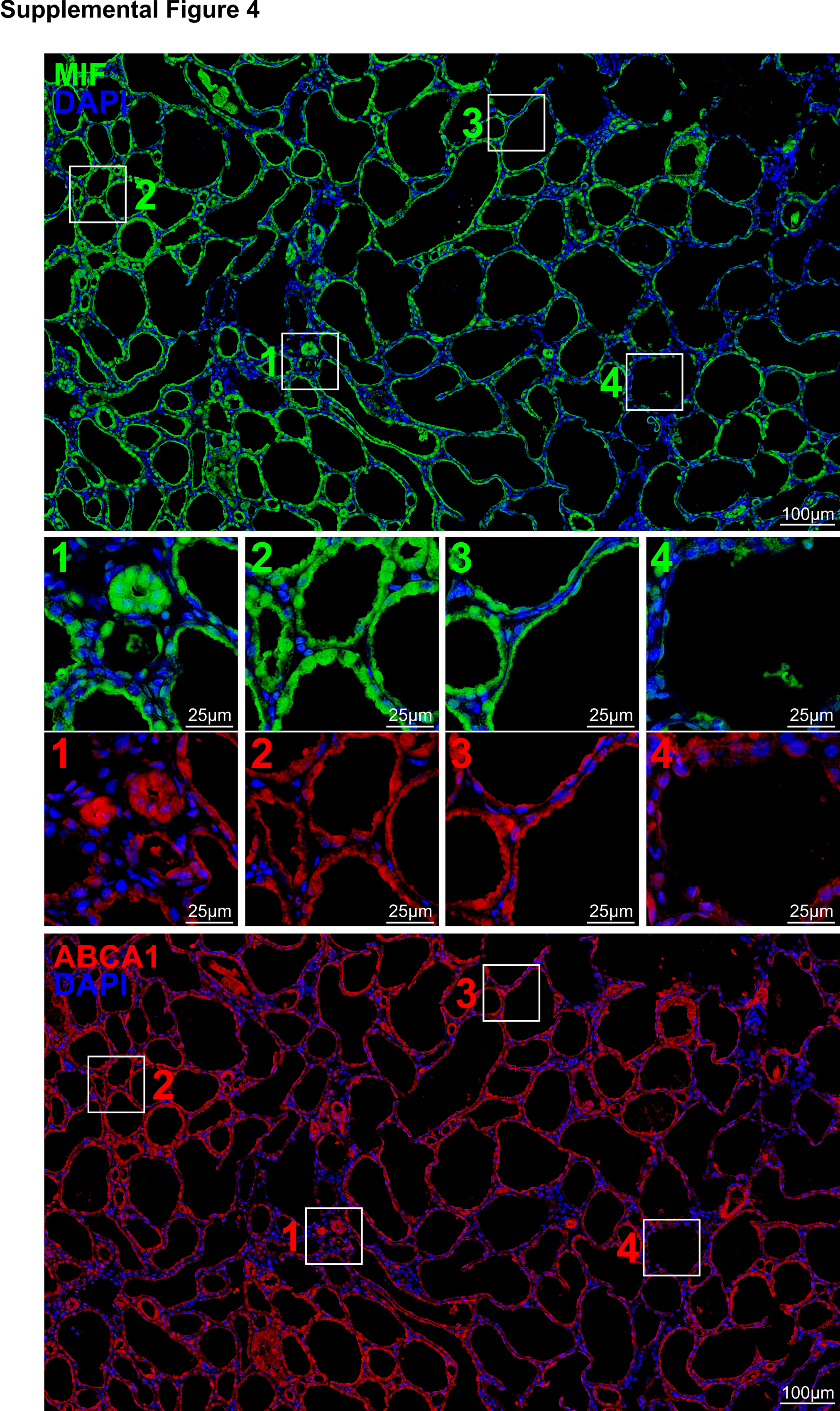

Supplement: Supplementary file 7 — MIF and ABCA1 are coexpressed in cyst-lining cells in vivo. Since ABCA1 has been shown to act as a transport protein for MIF, we stained serial sections of kidneys from KspCreERT2;Pkd1lox;lox mice treated with ICA for ABCA1 or MIF, respectively, in order to test for co-expression of ABCA1 (red) and MIF (green). Large fields of view of kidney sections confirm distinct co-expression of both proteins. Areas within the white squares numbered from 1 to 4 were magnified to further illustrate ABCA1- and MIF coexpression within representative cyst-lining cells. (1) shows a normal tubule with nuclear MIF expression and coexpression of ABCA1. (2) shows small cysts with a more cytoplasmic expression of MIF and coexpression with ABCA1. (3) shows a large cyst with cytoplasmic MIF expression and ABCA1 coexpression. (4) shows one of the few MIF-negative cysts which does also not express ABCA1 (PNG 4714 kb) [file 109_2020_1964_Fig10_ESM.png]

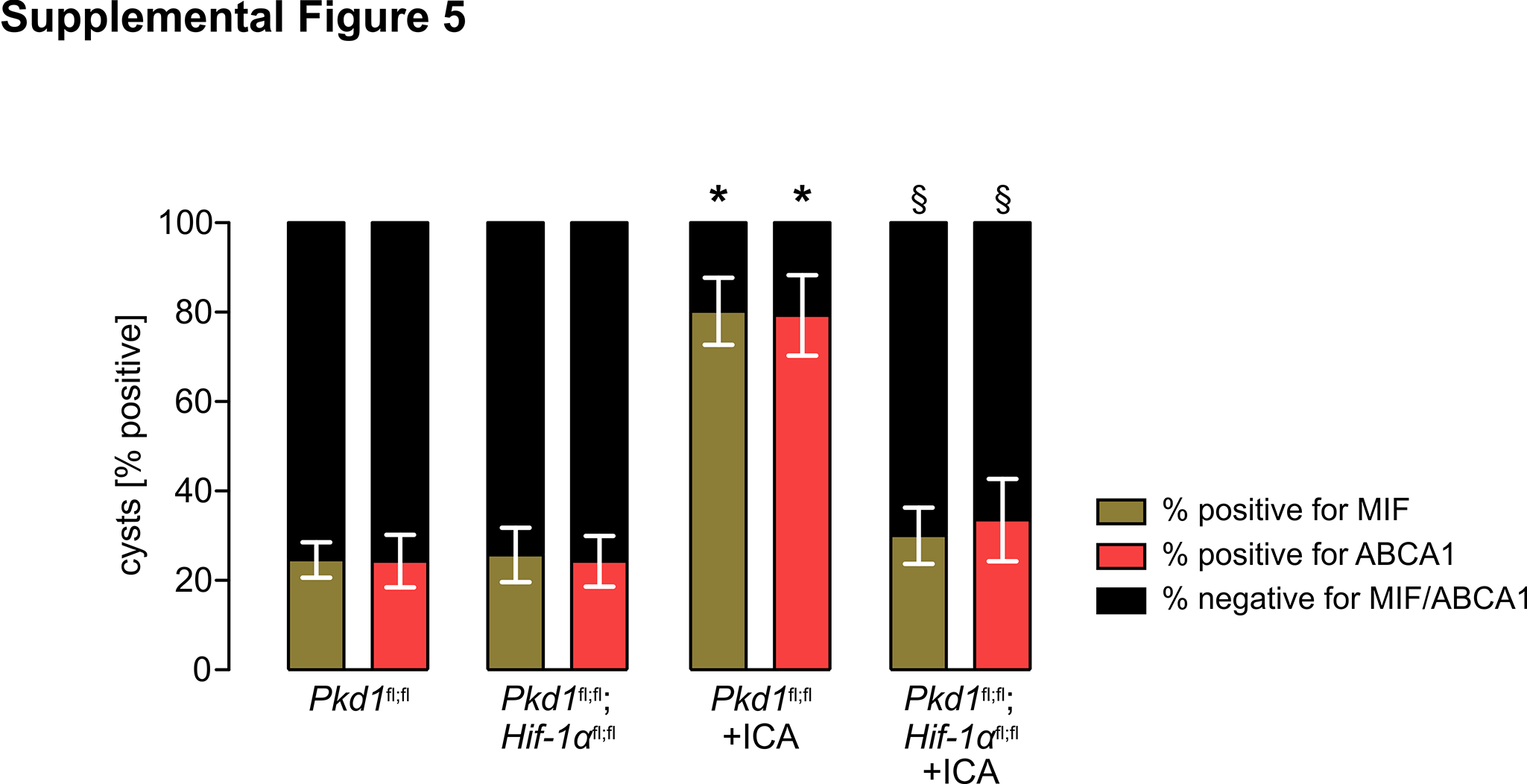

Supplement: Supplementary file 9 — The fraction of MIF- and ABCA1-positive cysts depends on HIF-1α in the ADPKD mouse model. In addition to the analyses in Fig. 1, the fraction of MIF- and ABCA1-positive cysts were analyzed in Pkd1fl;fl (n = 1708 cysts from n = 6 mice), Pkd1fl;fl;Hif-1αfl;fl (n = 1618 cysts from n = 6 mice), Pkd1fl;fl + ICA (n = 1319 cysts from n = 6 mice) and Pkd1fl;fl;Hif-1αfl;fl + ICA (n = 1469 cysts from n = 6 mice) mice. *Significant compared with Pkd1fl;fl. §Significant compared with Pkd1fl;fl + ICA (PNG 117 kb) [file 109_2020_1964_Fig11_ESM.png]

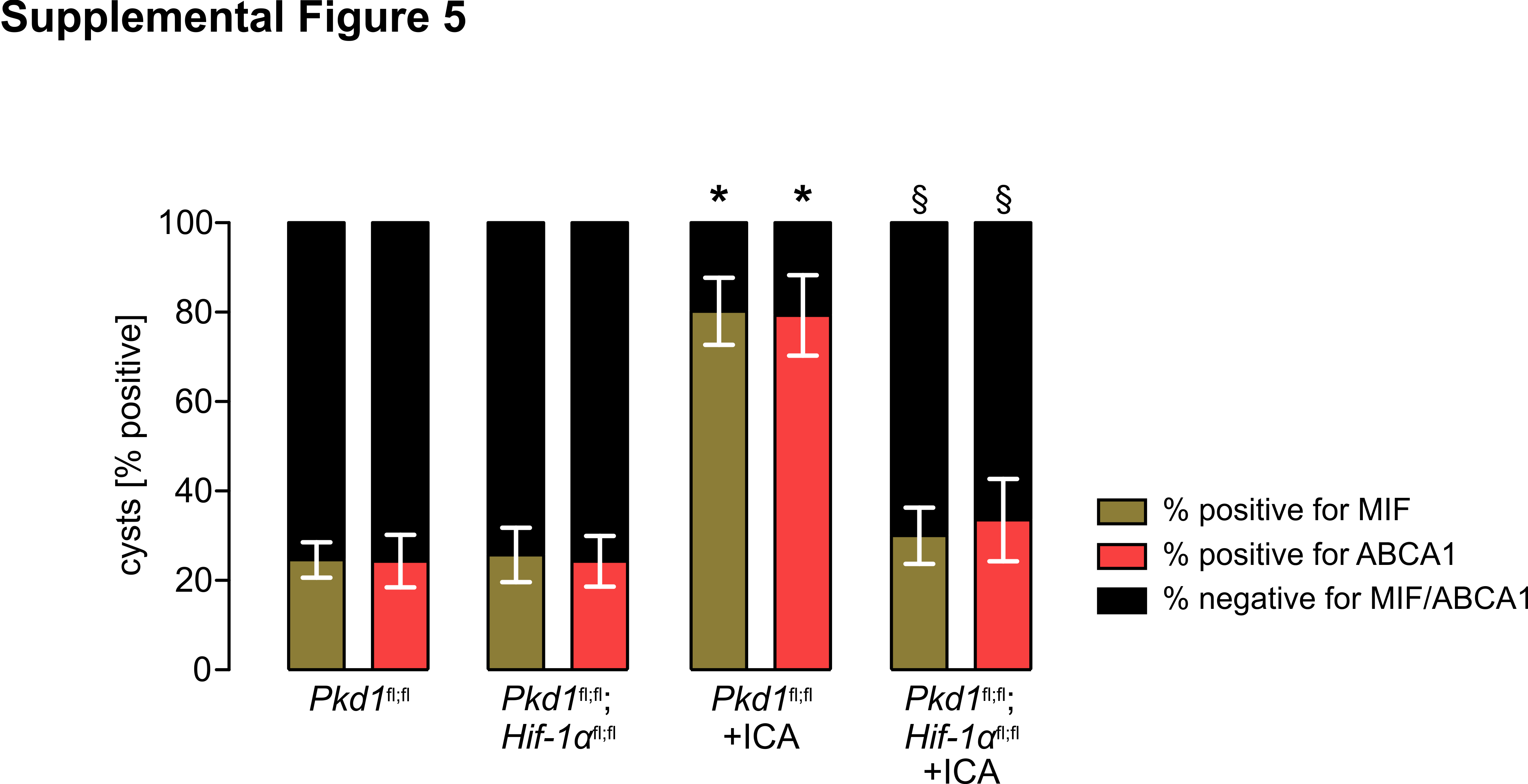

Supplement: Supplementary file 10 — High resolution image (TIF 25292 kb) [file 109_2020_1964_MOESM5_ESM.tif]

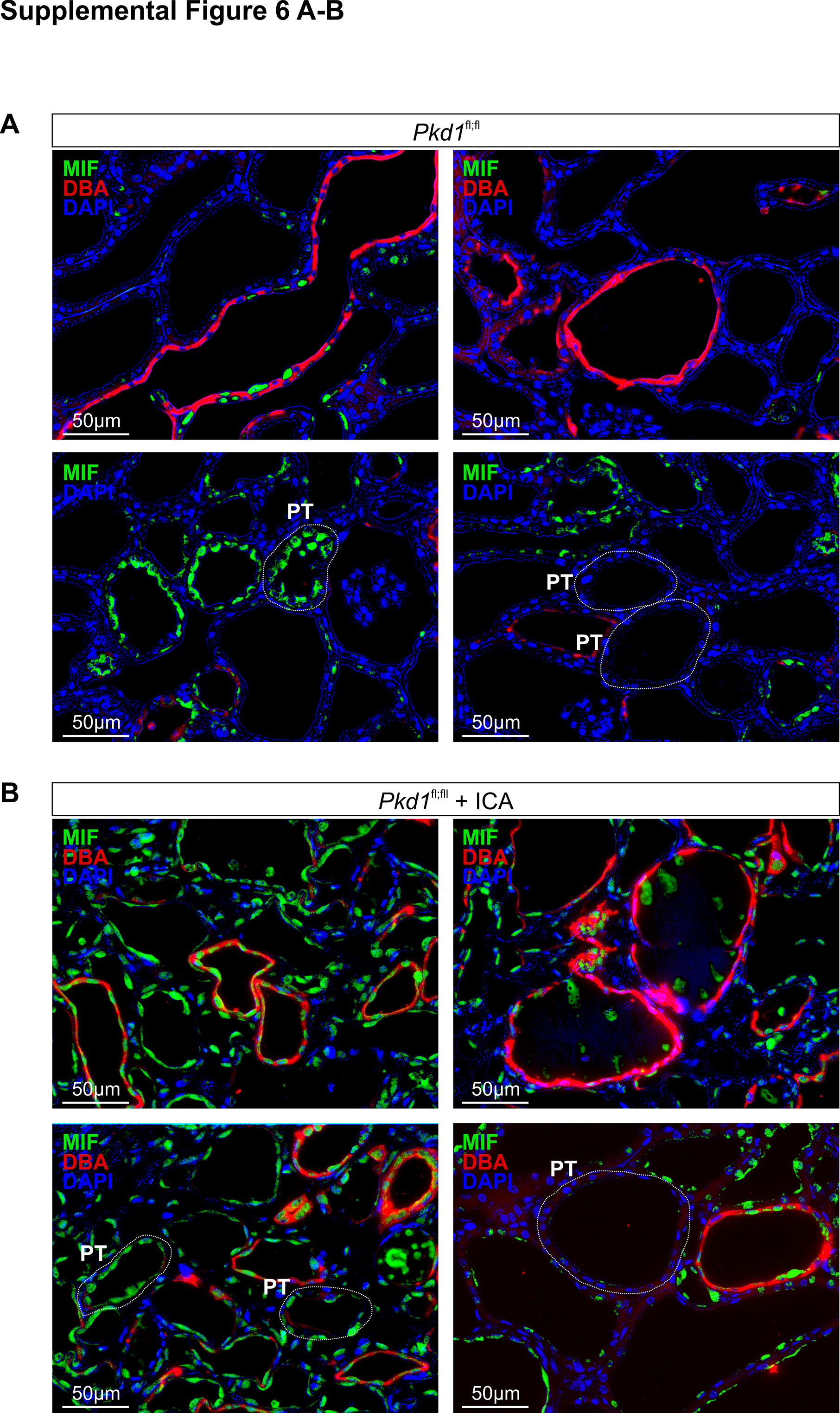

Supplement: Supplementary file 11 — MIF expression is not limited to specific tubular segments but correlates with expression of HIF-1α. A Pkd1fl;fl kidneys were stained for MIF (green) and nuclei (DAPI; blue) and analyzed for segment-specific expression. Upper row, left: MIF can be found in collecting ducts stained by dolichos biflorus agglutinin (DBA; red). Right: Collecting ducts can also be negative for MIF. Lower row, left: Proximal tubules (marked by white lines) can be stained positive for MIF, but also negative (right). B Kidneys from Pkd1fl;fl mice treated with ICA also show collecting ducts that are positive for MIF (upper row, left), but also a few that are negative (right). Lower row left shows proximal tubules that are stained positive for MIF. Right shows proximal tubule that is stained negative for MIF. C Kidneys from Pkd1fl;fl mice treated with ICA were stained for MIF (green), nuclei (DAPI; blue) and HIF-1α (red). Left photos show cysts expressing HIF-1α and to a great extent co-expression of MIF in serial sections. Right shows HIF-1α-negative cysts and for the most part absence of MIF expression. D Kidneys from Pkd1fl;fl;Hif-1αfl;fl mice treated with ICA were stained for MIF (green), nuclei (DAPI; blue) and HIF-1α (red). Left photos show typical cysts lacking HIF-1α and also MIF in serial sections. Right shows one of few HIF-1α-positive cysts and to a great extent coexpression of MIF (PNG 3252 kb) [file 109_2020_1964_Fig12_ESM.png]

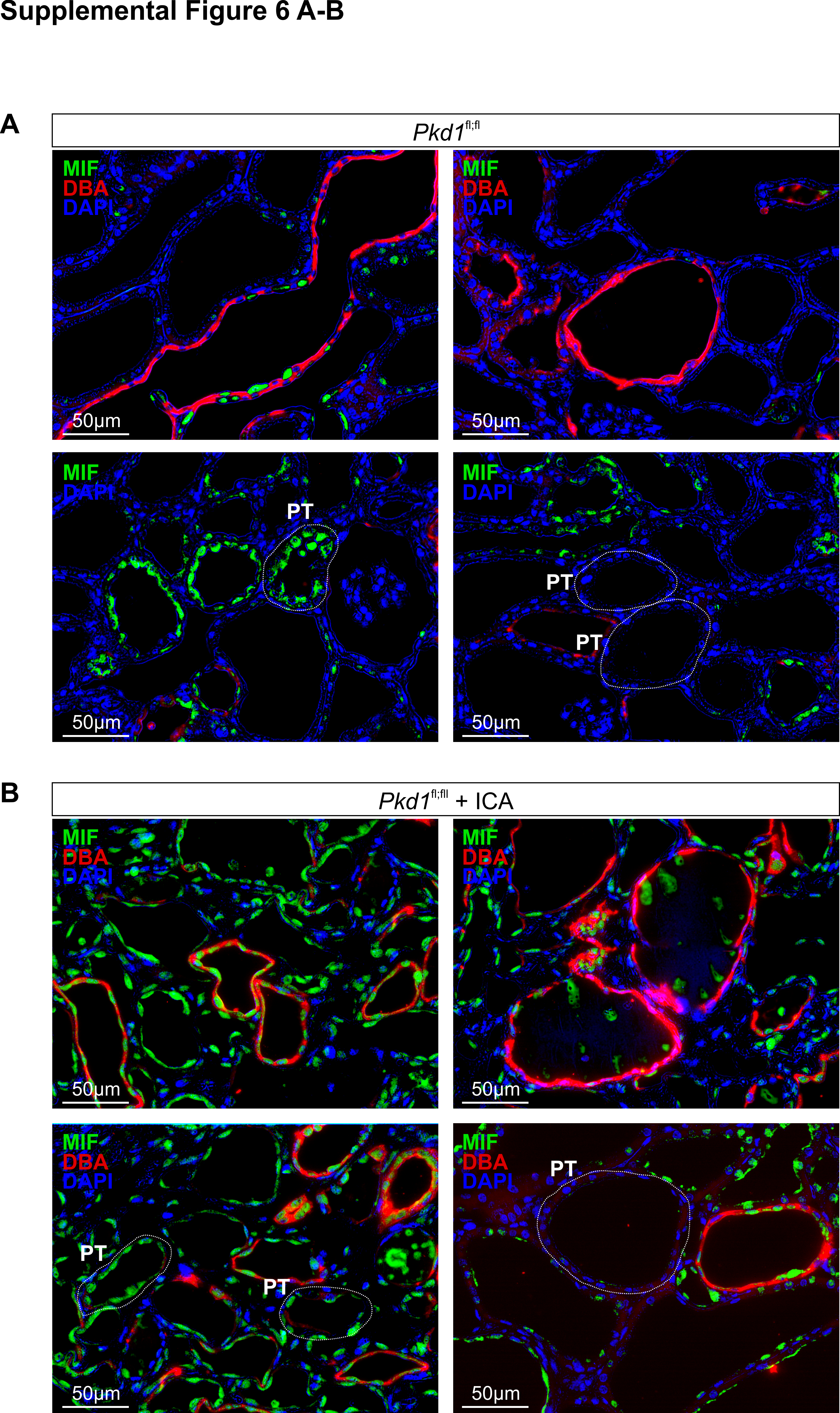

Supplement: Supplementary file 12 — High resolution image (TIF 64758 kb) [file 109_2020_1964_MOESM6_ESM.tif]

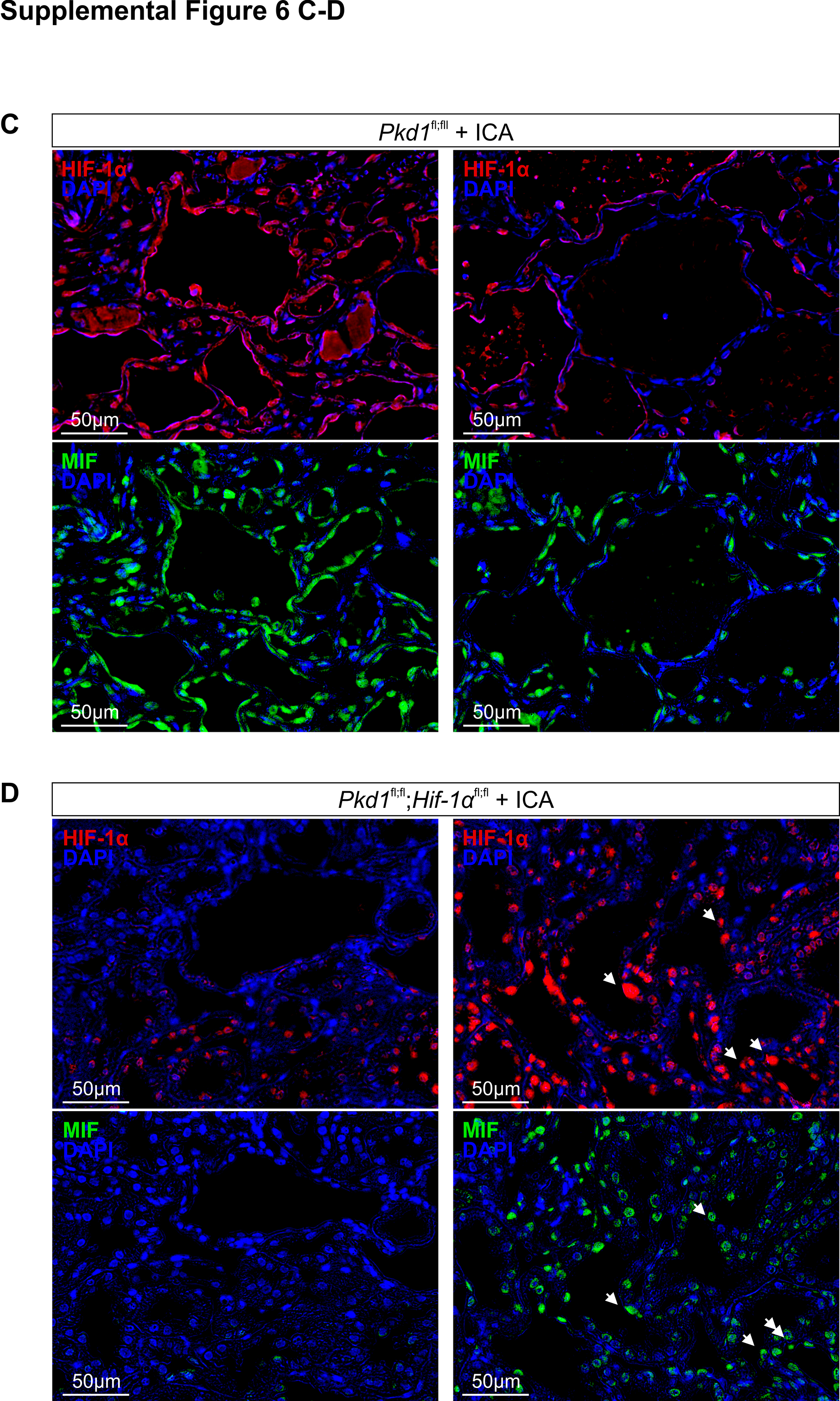

Supplement: Supplementary file 13 — (PNG 2824 kb) [file 109_2020_1964_Fig13_ESM.png]

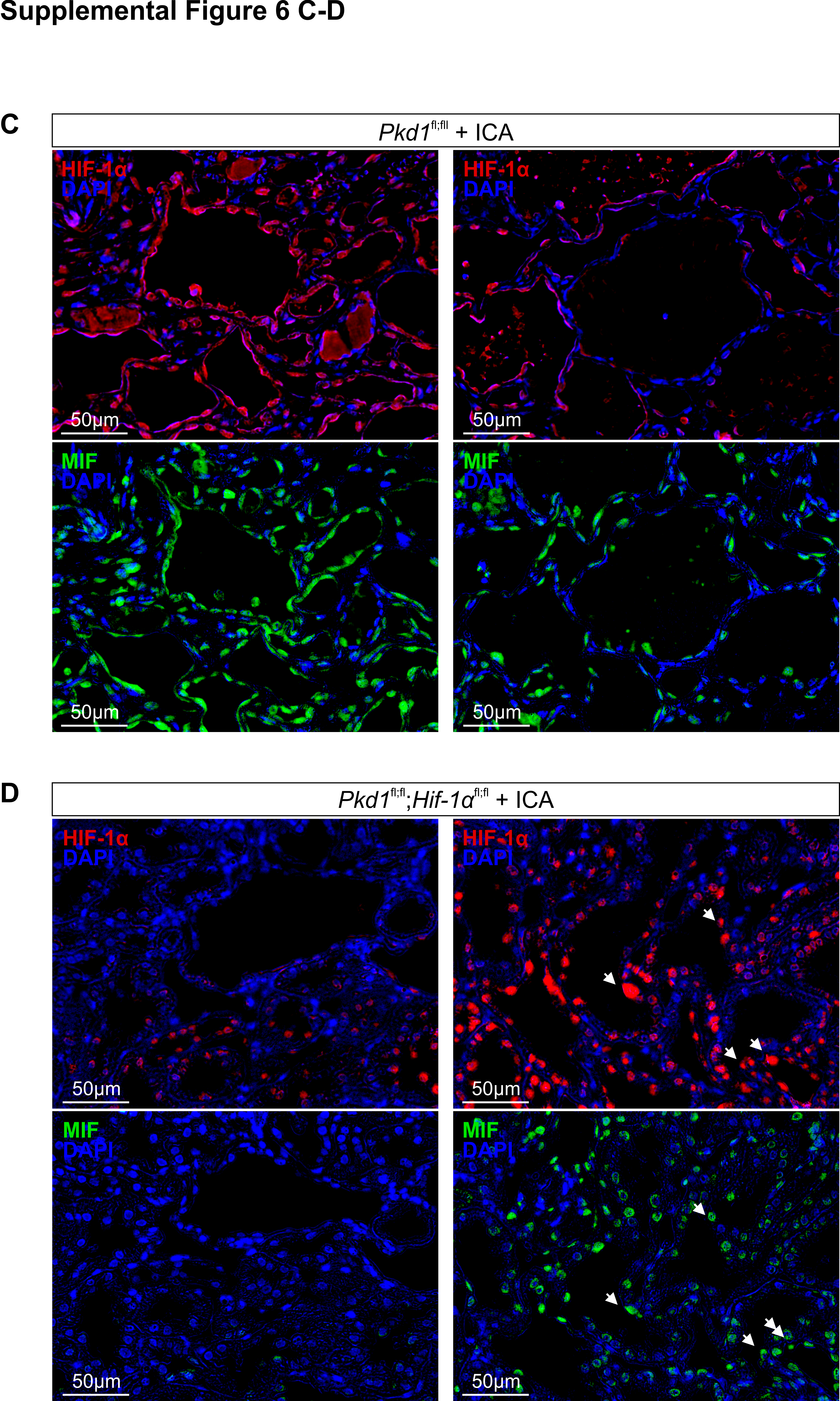

Supplement: Supplementary file 14 — High resolution image (TIF 64206 kb) [file 109_2020_1964_MOESM7_ESM.tif]

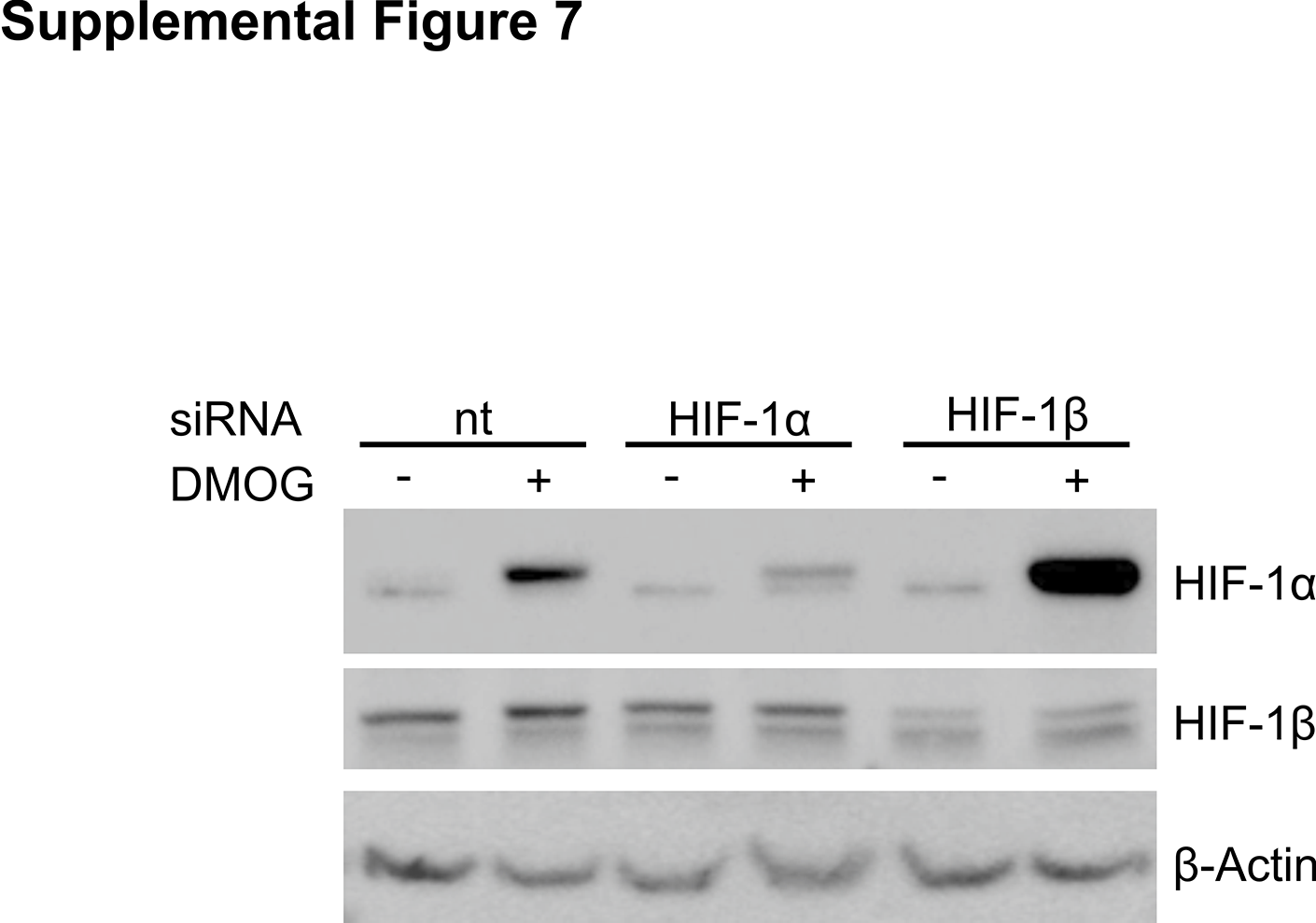

Supplement: Supplementary file 15 — HIF protein is reduced in hPTCs by siRNA directed against HIF-1α or HIF-1β. Human primary renal tubular cells were either treated with siRNA directed against HIF-1α or HIF-1β, respectively and stimulated with DMOG for 16 hours. Representative western blot showing reduced levels of HIF-1α or HIF-1β on protein level upon application of siRNA directed against HIF-1α or HIF-1β, respectively (PNG 183 kb) [file 109_2020_1964_Fig14_ESM.png]

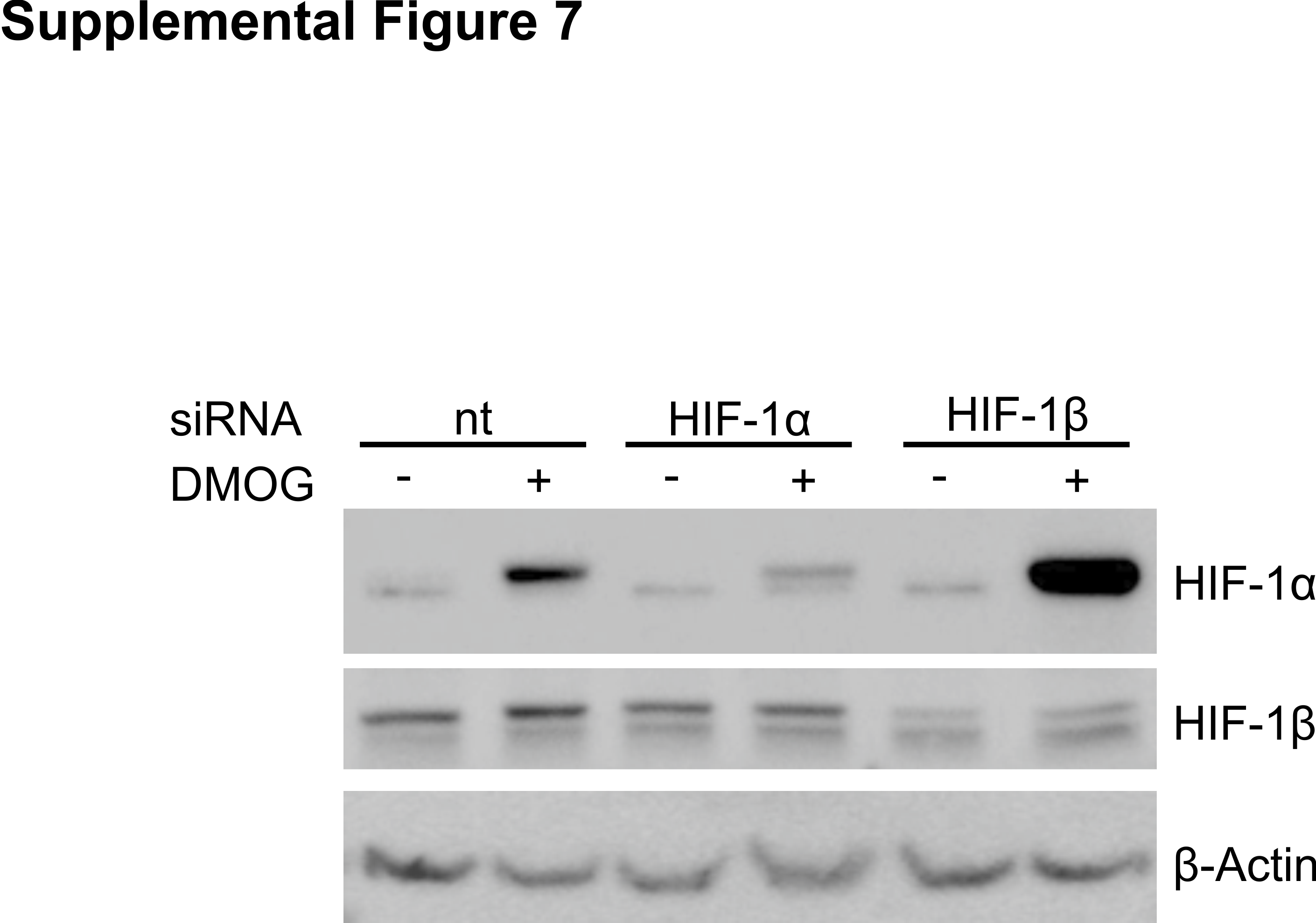

Supplement: Supplementary file 16 — High resolution image (TIF 16359 kb) [file 109_2020_1964_MOESM8_ESM.tif]

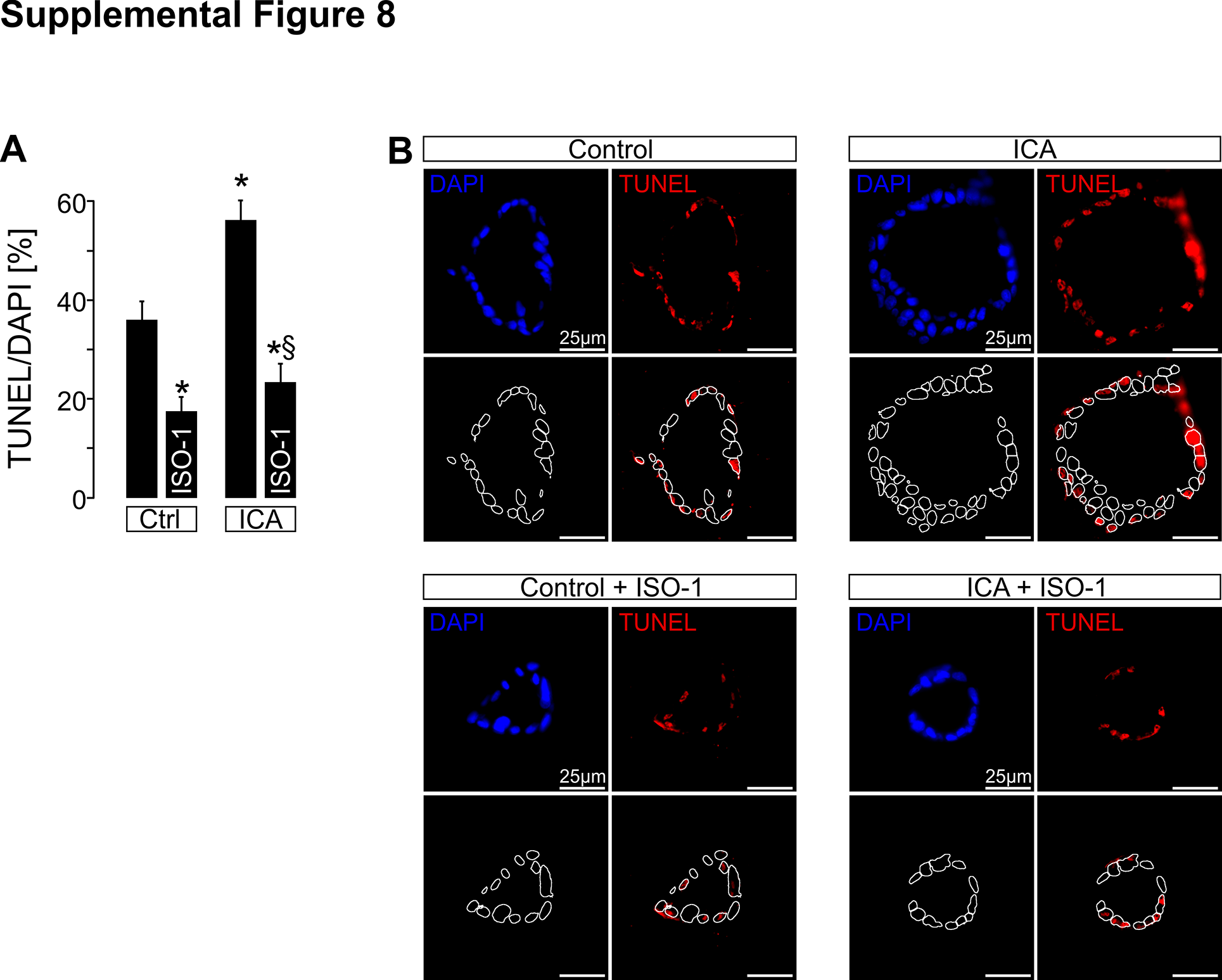

Supplement: Supplementary file 17 — Inhibition of MIF results in reduction of in vitro cyst cell apoptosis. plMDCK cells forming cysts were exposed to control medium containing forskolin (10 μM) ± application of the MIF-inhibitor ISO-1 (10 μM) and ± application of ICA (10 μM) for 5 days. A Quantification of TUNEL-positive cells in the cysts (n = 120-130 cysts per condition) normalized to total cyst cell number obtained by DAPI staining. B Representative stainings of cysts for nuclei (DAPI; blue) and TUNEL (red). Outline of nuclei stained by DAPI were marked by a white line to better visualize the fraction of TUNEL-positive cells and total cell number by overlay (lower rows). *Significant compared with Ctrl. § significant compared to ICA (PNG 291 kb) [file 109_2020_1964_Fig15_ESM.png]

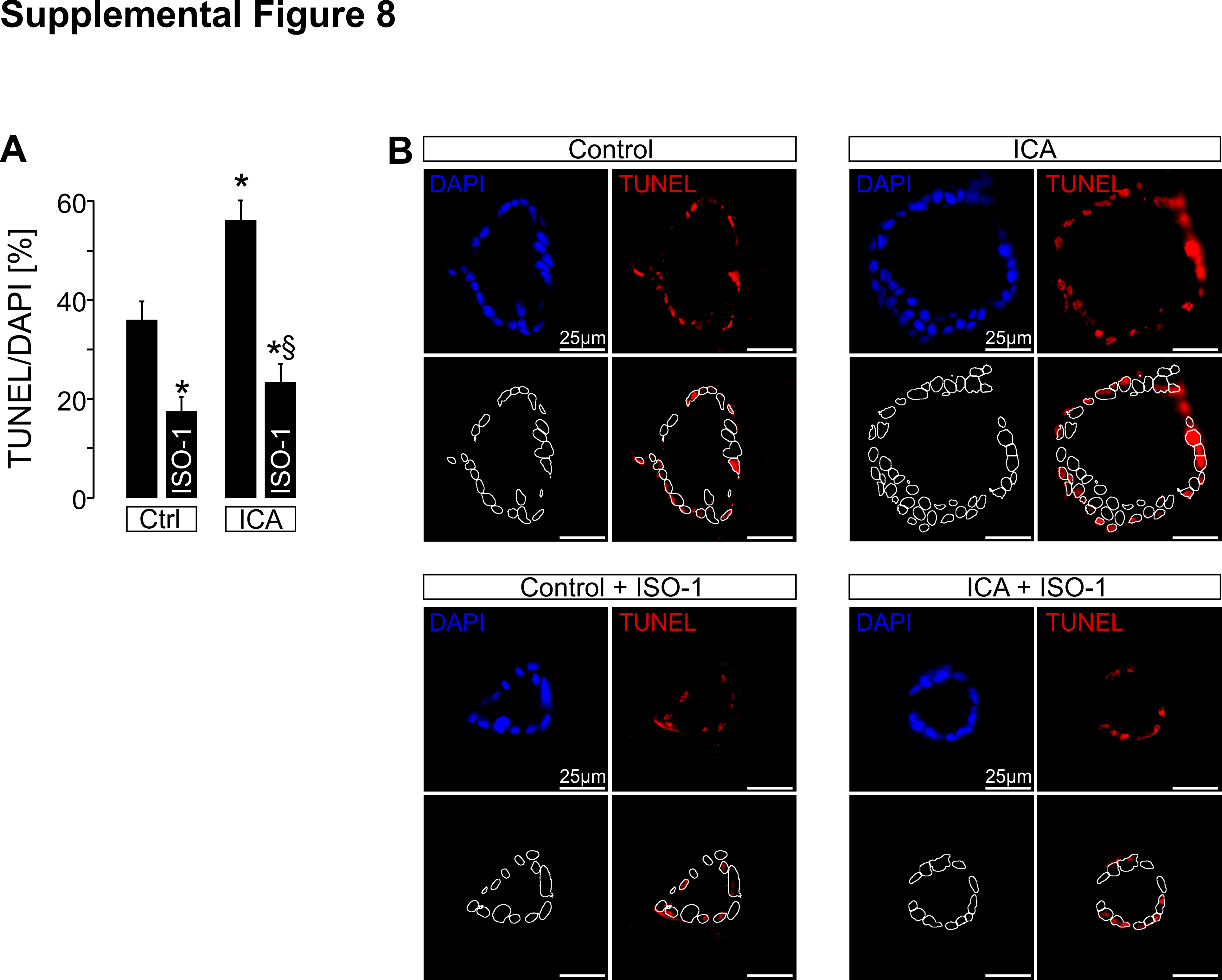

Supplement: Supplementary file 18 — High resolution image (TIF 34995 kb) [file 109_2020_1964_MOESM9_ESM.tif]
