# Supplementary material for: Macrophage migration inhibitory factor is regulated by HIF-1α and cAMP and promotes renal cyst cell proliferation in a macrophage-independent manner
Source: J Mol Med (Berl). 2020 Sep 4;98(11):1547–59. doi: 10.1007/s00109-020-01964-1 (PMC7591438; doi:10.1007/s00109-020-01964-1)
Supplement: Supplementary file 19 — (DOCX 13 kb) [file 109_2020_1964_MOESM10_ESM.docx]

**Supplementary Table 1: Primer sequences**

| Gene | Species | Forward 5´-3´ | Reverse 5´-3´ |
| --- | --- | --- | --- |
| HPRT | human | GACCAGTCAACAGGGGACAT | AACACTTCGTGGGGTCCTTTTC |
| MIF | human | CATCGTAAACACCAACGTGC | GAAGGCCATGAGCTGGTC |
| ABCA1 | human | CCTGATCTCTGTTCGGCTGA | CCCTGAACCCAAGGAAGTGT |
| EGLN3 | human | GGCCATCAGCTTCCTCCTG | GGTGATGCAGCGACCATCA |
